# Supplementary material for: An analytical process of spatial autocorrelation functions based on Moran’s index
Source: PLoS One. 2021 Apr 14;16(4):e0249589. doi: 10.1371/journal.pone.0249589 (PMC8046215; doi:10.1371/journal.pone.0249589)
Supplement: Attachment — Submitted filename: PONE-D-20-34327_reviewerA.pdf [file pone.0249589.s004.pdf]

PLOS ONE

An Analytical Process of Spatial Autocorrelation Functions Based on Moran's Index

--Manuscript Draft--

**Manuscript Number:**	PONE-D-20-34327
**Article Type:**	Research Article
**Full Title:**	An Analytical Process of Spatial Autocorrelation Functions Based on Moran's Index
**Short Title:**	Spatial Autocorrelation Function Analyses
**Corresponding Author:**	Yanguang Chen, Ph.D<br>Peking University<br>Beijing, CHINA
**Keywords:**	spatial autocorrelation function (SACF); spatial partial autocorrelation function (SPACF); Moran's index; Geary's coefficient; Getis-Ord's index; spatial weights matrix; spatial analysis; spatial modeling
**Abstract:**	A number of spatial statistic measurements such as Moran's I and Geary's C can be used to make spatial autocorrelation analysis. The spatial autocorrelation modeling proceeded from the 1-dimension autocorrelation of time series analysis, however, the time lag has been replaced by spatial weights so that the autocorrelation functions degenerated to autocorrelation coefficients. This paper is devoted to developing 2-dimensional spatial autocorrelation functions based on Moran's index. Using the relative staircase function as a weight function to yield a spatial weight matrix with displacement parameter. The displacement bears analogy with time lag of time series analysis. Based on the spatial displacement parameter, two types of spatial autocorrelation functions are constructed for 2-dimensional spatial analysis. Then the spatial autocorrelation functions are generalized to the autocorrelation functions based on Geary's coefficient and Getis' index. As an example, the new analytical framework are applied to the spatial autocorrelation modeling of Chinese cities. A conclusion can be reached that it is an effective method to build spatial autocorrelation function based on relative step function. The spatial autocorrelation functions can be employed to reveal deep geographical information and seek characteristic scales, and lay the foundation for the scaling analysis of spatial correlation.
**Order of Authors:**	Yanguang Chen, Ph.D
**Additional Information:**	
**Question**	**Response**
**Financial Disclosure**<br><br>Enter a financial disclosure statement that describes the sources of funding for the work included in this submission. Review the submission guidelines for detailed requirements. View published research articles from *PLOS ONE* for specific examples.<br><br>This statement is required for submission and **will appear in the published article** if the submission is accepted. Please make sure it is accurate.	The funders had no role in study design, data collection and analysis, decision to publish, or preparation of the manuscript.

Powered by Editorial Manager® and ProduXion Manager® from Aries Systems Corporation

**Unfunded studies**<br>Enter: *The author(s) received no specific funding for this work.*<br><br>**Funded studies**<br>Enter a statement with the following details:<br>• Initials of the authors who received each award<br>• Grant numbers awarded to each author<br>• The full name of each funder<br>• URL of each funder website<br>• Did the sponsors or funders play any role in the study design, data collection and analysis, decision to publish, or preparation of the manuscript?<br>• **NO** - Include this sentence at the end of your statement: *The funders had no role in study design, data collection and analysis, decision to publish, or preparation of the manuscript.*<br>• **YES** - Specify the role(s) played.<br><br>* typeset	
**Competing Interests**<br><br>Use the instructions below to enter a competing interest statement for this submission. On behalf of all authors, disclose any competing interests that could be perceived to bias this work—acknowledging all financial support and any other relevant financial or non-financial competing interests.<br><br>This statement **will appear in the published article** if the submission is accepted. Please make sure it is accurate. View published research articles from *PLOS ONE* for specific examples.	The authors have declared that no competing interests exist.

Powered by Editorial Manager® and ProduXion Manager® from Aries Systems Corporation

**NO authors have competing interests**<br><br>Enter: *The authors have declared that no competing interests exist.*<br><br>**Authors with competing interests**<br><br>Enter competing interest details beginning with this statement:<br><br>*I have read the journal's policy and the authors of this manuscript have the following competing interests: [insert competing interests here]*<br><br>* typeset	
**Ethics Statement**<br><br>Enter an ethics statement for this submission. This statement is required if the study involved:<br><br>• Human participants<br>• Human specimens or tissue<br>• Vertebrate animals or cephalopods<br>• Vertebrate embryos or tissues<br>• Field research<br><br>Write "N/A" if the submission does not require an ethics statement.<br><br>General guidance is provided below. Consult the submission guidelines for detailed instructions. **Make sure that all information entered here is included in the Methods section of the manuscript.**	N/A

Powered by Editorial Manager® and ProduXion Manager® from Aries Systems Corporation

**Format for specific study types**<br><br>**Human Subject Research (involving human participants and/or tissue)**<br>• Give the name of the institutional review board or ethics committee that approved the study<br>• Include the approval number and/or a statement indicating approval of this research<br>• Indicate the form of consent obtained (written/oral) or the reason that consent was not obtained (e.g. the data were analyzed anonymously)<br><br>**Animal Research (involving vertebrate animals, embryos or tissues)**<br>• Provide the name of the Institutional Animal Care and Use Committee (IACUC) or other relevant ethics board that reviewed the study protocol, and indicate whether they approved this research or granted a formal waiver of ethical approval<br>• Include an approval number if one was obtained<br>• If the study involved *non-human primates*, add *additional details* about animal welfare and steps taken to ameliorate suffering<br>• If anesthesia, euthanasia, or any kind of animal sacrifice is part of the study, include briefly which substances and/or methods were applied<br><br>**Field Research**<br><br>Include the following details if this study involves the collection of plant, animal, or other materials from a natural setting:<br>• Field permit number<br>• Name of the institution or relevant body that granted permission	
**Data Availability**<br><br>Authors are required to make all data underlying the findings described fully available, without restriction, and from the time of publication. PLOS allows rare exceptions to address legal and ethical concerns. See the PLOS Data Policy and FAQ for detailed information.	Yes - all data are fully available without restriction

A Data Availability Statement describing where the data can be found is required at submission. Your answers to this question constitute the Data Availability Statement and **will be published in the article**, if accepted.<br><br>**Important:** Stating 'data available on request from the author' is not sufficient. If your data are only available upon request, select 'No' for the first question and explain your exceptional situation in the text box.<br><br>Do the authors confirm that all data underlying the findings described in their manuscript are fully available without restriction?	
**Describe where the data may be found in full sentences. If you are copying our sample text, replace any instances of XXX with the appropriate details.**<br><br>• If the data are **held or will be held in a public repository**, include URLs, accession numbers or DOIs. If this information will only be available after acceptance, indicate this by ticking the box below. For example: *All XXX files are available from the XXX database (accession number(s) XXX, XXX.).*<br>• If the data are all contained **within the manuscript and/or Supporting Information files**, enter the following: *All relevant data are within the manuscript and its Supporting Information files.*<br>• If neither of these applies but you are able to provide **details of access elsewhere**, with or without limitations, please do so. For example:<br><br>*Data cannot be shared publicly because of [XXX]. Data are available from the XXX Institutional Data Access / Ethics Committee (contact via XXX) for researchers who meet the criteria for access to confidential data.*<br><br>*The data underlying the results presented in the study are available from (include the name of the third party*	All the data can be found in the Supporting Information files.

and contact information or URL).<br>• This text is appropriate if the data are owned by a third party and authors do not have permission to share the data.<br><br>* typeset	
Additional data availability information:	Tick here if your circumstances are not covered by the questions above and you need the journal’s help to make your data available.

Manuscript

Click here to access/download;Manuscript;Manuscript for review 2020-11-01.docx

An Analytical Process of Spatial Autocorrelation Functions Based on Moran's Index

Yanguang Chen

(Department of Geography, College of Urban and Environmental Sciences, Peking University, 100871, Beijing, China. Email: chenyg@pku.edu.cn)

Abstract: A number of spatial statistic measurements such as Moran's *I* and Geary's *C* can be used to make spatial autocorrelation analysis. The spatial autocorrelation modeling proceeded from the 1-dimension autocorrelation of time series analysis, however, the time lag has been replaced by spatial weights so that the autocorrelation functions degenerated to autocorrelation coefficients. This paper is devoted to developing 2-dimensional spatial autocorrelation functions based on Moran's index. Using the relative staircase function as a weight function to yield a spatial weight matrix with displacement parameter. The displacement bears analogy with time lag of time series analysis. Based on the spatial displacement parameter, two types of spatial autocorrelation functions are constructed for 2-dimensional spatial analysis. Then the spatial autocorrelation functions are generalized to the autocorrelation functions based on Geary's coefficient and Getis' index. As an example, the new analytical framework are applied to the spatial autocorrelation modeling of Chinese cities. A conclusion can be reached that it is an effective method to build spatial autocorrelation function based on relative step function. The spatial autocorrelation functions can be employed to reveal deep geographical information and seek characteristic scales, and lay the foundation for the scaling analysis of spatial correlation.

Key words: spatial autocorrelation function (SACF); spatial partial autocorrelation function (SPACF); Moran's index; Geary's coefficient; Getis-Ord's index; spatial weights matrix; spatial analysis; spatial modeling

1 Introduction

Spatial autocorrelation is one of important methods of quantitative analyses in geography. This method can be treated as the cornerstone of spatial statistics. However, present spatial autocorrelation analysis has two significant shortcomings, which hinder its application scope and effect. First, in the theoretical aspect, the scaling property of geographical spatial distributions has been ignored ~~for a long time.~~ Conventional mathematical modeling and quantitative analysis depend on characteristic scales. If and only if we find the valid characteristic scales such as determinate length, eigenvalue, and mean, ~~we~~ will be able to make effective mathematical models. If a geographical distribution is a scale-free distribution, no characteristic scale can be found, and the conventional mathematical methods will be ineffective. In this case, the mathematical tools based on characteristic scales should be replaced by those based on scaling analysis (Chen, 2013a). Second, in the methodological aspect, the spatial displacement parameter has been neglected ~~for a long term.~~ Generalizing Pearson's simple cross-correlation coefficient to time series analysis yielded 1-dimensional temporal auto-correlation function (TACF) based on time lag parameter (Bowerman and O'Connell, 1993; Box *et al*, 1994). Generalizing temporal auto-correlation function (TACF) to ordered space series and substituting the time lag with spatial displacement yielded 1-dimensional spatial auto-correlation function (SACF) (Chen, 2013a). Generalizing the 1-dimensional spatial auto-correlation function to 2-dimensional spatial dataset and replacing the displacement parameters with spatial weight matrix yielded a 2-dimensional spatial autocorrelation coefficient, which is termed Moran's index, or Moran's I for short, in literature (Haggett *et al*, 1977). In principle, a time lag parameter corresponds to a spatial displacement parameter, which in turn corresponds to a weight matrix. Where ~~the~~ 1-dimensional autocorrelation analysis is concerned, a series of time lag parameters correspond to a series of spatial displacement parameters. However, only one spatial weight matrix can be taken into account in ~~the~~ conventional autocorrelation modeling.

~~If we~~ introduce a set of ordered spatial weight matrixes into the spatial autocorrelation models, ~~we will be able~~ to construct 2-dimensional spatial autocorrelation function. Based on the 2-dimensional autocorrelation function, spatial scaling analysis may be made ~~for~~ spatial correlation ~~processes~~. This paper ~~is devoted to~~ developing 2-dimensional spatial autocorrelation functions based on Moran's index and the corresponding analytical process, ~~and~~ laying the foundation for

scaling analysis based on spatial autocorrelation. The ~~rest~~ parts are organized as follows. In Section 2, two types of spatial autocorrelation functions based on Moran's index are established by using the relative staircase function as a weight function. In Section 3, the autocorrelation functions based on Moran's index are generalized to the autocorrelation functions based on Geary's coefficient and Getis's index. In Section 4, empirical analyses are made to show how to utilize the spatial autocorrelation functions to make analytical analyses of geographical phenomena. Finally, the main points of this study are summarized. The chief academic contribution of this paper is to extend the spatial autocorrelation coefficient to the spatial autocorrelation function, thus developing ~~the~~ spatial analysis method.

2 Theoretical results

2.1 Simplified expression of Moran's index

The first measurement of spatial autocorrelation ~~is~~ the well-known Moran's index, which is in fact a spatial autocorrelation coefficient. The formula of Moran's index bears a complicated form, but the expression can be simplified by means of matrix and vector. The formulae and expressions are not new in this subsection, but they are helpful for us to understand the new mathematical process shown in next subsection. Suppose there are n elements (e.g., cities) in a system (e.g., a network of cities) which can be measured by a variable (e.g., city size), x. In literature, global Moran's index can be expressed as ~~below~~

$$I = \frac{n\sum_{i=1}^{n}\sum_{j=1}^{n}v_{ij}(x_i-\mu)(x_j-\mu)}{\sum_{i=1}^{n}\sum_{j=1}^{n}v_{ij}\sum_{i=1}^{n}(x_i-\mu)^2}, \tag{1}$$

where I denotes Moran's I, ~~i.e., the most common spatial autocorrelation coefficient,~~ x_i is a size measurement of the ith element in a geographical spatial system (i=1,2,…,n), μ represents the mean of x_i, v_{ij} refers to the elements in a spatial contiguity matrix (SCM), V. The symbols can be developed as follows

$$x = [x_1 \quad x_2 \quad \cdots \quad x_n]^{\mathrm{T}}, \tag{2}$$

$$\mu = \frac{1}{n}\sum_{i=1}^{n}x_i, \tag{3}$$

$$V=\begin{bmatrix} v_{11} & v_{12} & \cdots & v_{1n} \\ v_{21} & v_{22} & \cdots & v_{2n} \\ \vdots & \vdots & \ddots & \vdots \\ v_{n1} & v_{n2} & \cdots & v_{nn} \end{bmatrix}=[v_{ij}]_{n\times n}. \tag{4}$$

Using ~~the knowledge of~~ algebra, we can simplify the formula of Moran's index and re-express it as below (Chen, 2013b)

$$I=z^{\mathrm{T}}Wz, \tag{5}$$

in which z denotes the standardized size vector based on population standard deviation, W is the unitized spatial contiguity matrix (USCM), i.e., a spatial weight matrix (SWM), the superscript T indicates matrix or vector transposition. The standardized size vector is as follows

$$z=\frac{x-\mu}{\sigma}, \tag{6}$$

where σ refers to population standard deviation, which can be expressed as

$$\sigma=[\frac{1}{n}\sum_{i=1}^{n}(x_i-\mu)^2]^{1/2}=[\frac{1}{n}(x-\mu)^{\mathrm{T}}(x-\mu)]^{1/2}. \tag{7}$$

The spatial weight matrix, W, can be expressed as

$$W=\frac{V}{V_0}=\begin{bmatrix} w_{11} & w_{12} & \cdots & w_{1n} \\ w_{21} & w_{22} & \cdots & w_{2n} \\ \vdots & \vdots & \ddots & \vdots \\ w_{n1} & w_{n2} & \ddots & w_{nn} \end{bmatrix}=[w_{ij}]_{n\times n}, \tag{8}$$

where

$$V_0=\sum_{i=1}^{n}\sum_{j=1}^{n}v_{ij}, \tag{9}$$

represents the summation of the numeric value of matrix elements, and

$$w_{ij}=\frac{v_{ij}}{V_0}=\frac{v_{ij}}{\sum_{i=1}^{n}\sum_{j=1}^{n}v_{ij}}, \tag{10}$$

denotes the unitized value of the ith row and the jth column in the weight matrix. Apparently, the matrix W satisfies the following relation

$$\sum_{i=1}^{n}\sum_{j=1}^{n}w_{ij}=1, \tag{11}$$

which is termed normalization condition and W is termed normalization matrix in literature (Chen, 2013b). In fact, the normalization process is based on range, namely, the difference between the

maximum and minimum values, rather than based on summation (Chen, 2012). Besides the unitization indicated by equation (11), the matrix has another two characteristics. One is symmetry, i.e., $w_{ij}=w_{ji}$; the other is zero diagonal elements, namely, $|w_{ii}|=0$, which implies no self-correlation of an element itself. Spatial contiguity matrix comes from spatial distance matrix, which is a symmetric hollow matrix. The distance axioms influence the properties of spatial weight matri~~x~~es (Chen, 2016).

Scientific description always relies heavily on a characteristic scale of a geographical system. In mathematics, characteristic scales include radius, side length, eigenvalue, average values, and standard deviation. For a ~~transform~~ **T**, if a function $f(x)$ is an eigen function if it satisfies the following relation

$$\mathbf{T}(f(x)) = \lambda f(x), \tag{12}$$

where λ is the corresponding eigenvalue of the function. This relation can be generalized to matrix equations. It can be proved that Matrix's index is the eigenvalue of generalized spatial correlation matrixes. Based on the inner product of the standardized size vector, a Real Spatial Correlation Matrix (RSCM) can be defined as

$$M = nW = z^{\mathrm{T}}zW, \tag{13}$$

where $n=z^{\mathrm{T}}z$ represents the *inner product* of z. Thus we have

$$Mz = nWz = z^{\mathrm{T}}zWz = Iz, \tag{14}$$

which indicates that I is the largest characteristic root of the polynomial equation proceeding from the determinant of the matrix nW, and z is just the corresponding characteristic vector. Based on the outer product of z, ~~a~~ Ideal Spatial Correlation Matrix (ISCM) can be defined as

$$M^{*} = zz^{\mathrm{T}}W, \tag{15}$$

where zz^{T} represents the *outer product* of the standardized size vector. Then we have

$$M^{*}z = zz^{\mathrm{T}}Wz = Iz, \tag{16}$$

which implies that I is the largest eigen value the generalized spatial correlation matrix $zz^{\mathrm{T}}W$, and z is just the corresponding eigenvector of $zz^{\mathrm{T}}W$. This suggests that geographers have been taking advantage of the characteristic parameter ~~to make the~~ spatial analyses based on autocorrelation.

2.2 Standard spatial autocorrelation function based on Moran's *I*

The conventional mathematical modeling and quantitative analysis are based on characteristic scales. A good mathematical model of a system is usually involved with three scales, and thus includes three levels of parameters. The first is the macro-scale parameter indicating environmental level, the second is the micro-scale parameter indicating the element level, and the third is the characteristic scale indicating the key level (Hao, 1986). A characteristic scale is often expressed as a characteristic length due to it is always a 1-dimensional measure. In geometry, a characteristic length may be the radius of a circle or the side length of a square; in algebra, a characteristic length may be the eigen values of a square matrix or characteristic roots of a polynomial; in probability theory and statistics, a characteristic length may be the mean value and standard deviation of a probability distribution. As demonstrated above, Moran's index is the maximum eigen values of the generalized spatial correlation matrixes. Although the characteristic scales are expressed as radius, length of a side, eigenvalue, mean value, standard deviation, and so on, the reverse is not necessarily true. In other words, the radius, the side length, the eigenvalue, the mean value and the standard deviation do not necessarily represent a characteristic scale. If and if a quantity can be objectively determined and its value does not depend on the scale of measurement, the quantity can be used to represent a characteristic length. Can Moran's index be evaluated uniquely and objectively under given spatio-temporal conditions? This is still a pending question in theoretical and quantitative geographies, needing answer to it. To find the answer, we should calculate the Moran's index by means of different spatial scales.

Moran's index is a spatial autocorrelation coefficient (SACC), but it can be generalized to spatial autocorrelation function (SACF). A spatial autocorrelation function is a set of a series of ordered autocorrelation coefficients. The spatial autocorrelation function can be derived from the proper spatial weight functions. Four types of spatial weight functions can be used to generate spatial contiguity matrixes: inverse power function, negative exponential function, absolute staircase function, and relative staircase function (Chen, 2012; Chen, 2015; Chen, 2016; Cliff and Ord, 1973; Getis, 2009; Odland, 1988). Among these spatial weight function[illegible]only the relative staircase function can be used to construct a spatial autocorrelation function. A relative staircase function can be expressed as

$$v_{ij}(r)=f(r)=\begin{cases}1, & 0<d_{ij}\le r\\ 0, & d_{ij}>r, d_{ij}=0\end{cases}, \tag{17}$$

where d_{ij} denotes the distance between location *i* and *j*, and *r* represents the threshold value of spatial distance. In literature, the threshold value *r* is always represented by an average value, and is treated as a constant. However, a complex system often has no effective average value. In other word, complex systems are scale-free systems and have no characteristic scales. In this case, the quantitative analysis based on characteristic scale should be replaced by scaling analysis. In fact, spatial statisticians and theoretical geographers have aware the uncertainty of the threshold, *r* (Getis and Ord, 1992; Ord and Getis, 1995). Suppose that *r* is a variable rather than a constant. The spatial contiguity matrix, equation (4), should be rewritten as

$$V(r)=\begin{bmatrix} v_{11}(r) & v_{12}(r) & \cdots & v_{1n}(r)\\ v_{21}(r) & v_{22}(r) & \cdots & v_{2n}(r)\\ \vdots & \vdots & \ddots & \vdots\\ v_{n1}(r) & v_{n2}(r) & \cdots & v_{nn}(r)\end{bmatrix}=[v_{ij}(r)]_{n\times n}. \tag{18}$$

Accordingly, the spatial weight matrix, equation (8), can be re-expressed as

$$W(r)=\frac{V(r)}{V_0(r)}=\begin{bmatrix} w_{11}(r) & w_{12}(r) & \cdots & w_{1n}(r)\\ w_{21}(r) & w_{22}(r) & \cdots & w_{2n}(r)\\ \vdots & \vdots & \ddots & \vdots\\ w_{n1}(r) & w_{n2}(r) & \ddots & w_{nn}(r)\end{bmatrix}=[w_{ij}(r)]_{n\times n}, \tag{19}$$

where

$$V_0(r)=\sum_{i=1}^{n}\sum_{j=1}^{n}v_{ij}(r)=e^{\mathrm{T}}V(r)e, \tag{20}$$

$$w_{ij}(r)=\frac{v_{ij}(r)}{\sum_{i=1}^{n}\sum_{j=1}^{n}v_{ij}(r)}. \tag{21}$$

In equation (20), $e=[1\ 1\ \ldots\ 1]^{\mathrm{T}}$ refers to the special vector that including *n* elements, 1. The unitization property of spatial weight matrices remain unchanged, i.e.,

$$\sum_{i=1}^{n}\sum_{j=1}^{n}w_{ij}(r)=1. \tag{22}$$

The global spatial autocorrelation function (GSACF) can be defined as

$$I(r)=z^{\mathrm{T}}W(r)z, \tag{23}$$

which comes from the global Moran's index and relative staircase function, equations (5) and (17).

The distance threshold is a type of displacement parameter, which correspond to the time lag parameter in the temporal autocorrelation models of time series analysis. In this framework, Moran's index is no longer a spatial autocorrelation coefficient. It becomes a function of spatial displacement r. By means of the spatial autocorrelation function, we can make quantitative analyses of geographical spatial dynamics. The distance threshold can be defined as

$$r = kr_0 , \tag{24}$$

where k=1, 2, 3,…,m represent natural numbe[illegible]d r_0 is a constant. Empirically, we have $r_0 \geq \min(d_{ij})$ and $m \leq \max(d_{ij})/r_0$. This implies that the distance threshold comes between the minimum distance and the maximum distance, namely, $\min(d_{ij}) \leq r \leq \max(d_{ij})/r_0$.

2.3 Generalized spatial autocorrelation function based on Moran's *I*

In the above defined spatial autocorrelation function, each value represents an autocorrelation coefficient. In other word, if a distance threshold value r is given, then we have a standard Moran's index. Spatial autocorrelation analysis originated from time series analysis. However, this kind of autocorrelation function does not bear the same structure with the temporal autocorrelation function in time series analysis. If we construct a "weight matrix" to compute the autocorrelation function of a time series, the "weight matrix" is a quasi-unitized matrix instead of a strict unitized matrix. Actually, by analogy with the temporal autocorrelation function, we can improve the spatial autocorrelation function by revising the spatial weight matrix. The key lies in equation (20). According to the property of the spatial contiguity matrix based on the relative staircase function, the maximum of $V_0(r)$ is

$$\max(V_0(r)) = \lim_{r \to \max(d_{ij})} \sum_{i=1}^{n} \sum_{j=1}^{n} v_{ij}(r) = n(n-1) . \tag{25}$$

Thus the spatial weight matrix, equation (19), can be revised as

$$W^*(r) = \frac{1}{n(n-1)} V(r) = \begin{bmatrix} w_{11}^*(r) & w_{12}^*(r) & \cdots & w_{1n}^*(r) \\ w_{21}^*(r) & w_{22}^*(r) & \cdots & w_{2n}^*(r) \\ \vdots & \vdots & \ddots & \vdots \\ w_{n1}^*(r) & w_{n2}^*(r) & \ddots & w_{nn}^*(r) \end{bmatrix} = [w_{ij}^*(r)]_{n \times n} . \tag{26}$$

Based on equation (26), the spatial autocorrelation function can be re-defined as

$$I^*(r) = z^{\mathrm{T}} W^*(r) z = \frac{1}{n(n-1)} z^{\mathrm{T}} V(r) z, \tag{27}$$

which bears a strict analogy with the temporal autocorrelation function of time series analysis. The difference between equation (23) and equation (27) is as follows, for $I(r)$, $V_0(r)$ is a variable which depends on the distance threshold r, while for $I^*(r)$, $V_0(r)$ is a constant which is independent of r. In this case, the weight matrix does not always satisfy the unitization condition, and we have an inequality as below

$$\sum_{i=1}^{n}\sum_{j=1}^{n} w_{ij}^*(r) \le 1. \tag{28}$$

This implies that the summation of the elements in $W^*(r)$ is equal to or less than 1.

2.4 Partial spatial autocorrelation function based on Moran's *I*

Autocorrelation coefficients reflect both direct correlation and indirect correlation between the elements in a sample or spatial dataset. If we want to measure the pure direct autocorrelation and neglect the indirect autocorrelation, we should compute the partial autocorrelation coefficients. A set of ordered partial autocorrelation coefficients compose an autocorrelation function. In fact, we transform the spatial autocorrelation functions into the partial autocorrelation functions by means of the Yule-Walker recursive equation:

$$\begin{bmatrix} I_1 \\ I_2 \\ I_3 \\ \vdots \\ I_m \end{bmatrix} = \begin{bmatrix} 1 & I_1 & I_2 & \cdots & I_{m-1} \\ I_1 & 1 & I_1 & \cdots & I_{m-2} \\ I_2 & I_1 & 1 & \cdots & I_{m-3} \\ \vdots & \vdots & \vdots & \ddots & \vdots \\ I_{m-1} & I_{m-2} & I_{m-3} & \cdots & 1 \end{bmatrix} \cdot \begin{bmatrix} H_1 \\ H_2 \\ H_3 \\ \vdots \\ H_m \end{bmatrix}. \tag{29}$$

where I_k denotes the kth order autocorrelation coefficient, and the parameter H_k is the corresponding auto-regression coefficients. Yule-Walker equation associates autocorrelation and auto-regression equation. The last auto-regression coefficient, H_m, is equal to the mth order partial autocorrelation coefficient (k=1, 2, 3,…, m). If m=1, we have the first-order partial autocorrelation coefficient, which can be given by

$$[J_1] = [1] \cdot [H_1] = [H_1], \tag{30}$$

in which J_1=H_1 is the first-order partial autocorrelation coefficient. If m=2, we have the second-order partial autocorrelation coefficient, which can be given by the following matrix equation

$$\begin{bmatrix} I_1 \\ I_2 \end{bmatrix} = \begin{bmatrix} 1 & I_1 \\ I_1 & 1 \end{bmatrix} \cdot \begin{bmatrix} H_1 \\ H_2 \end{bmatrix}, \tag{31}$$

where $J_2=H_2$ is the second-order partial autocorrelation coefficient. If $m=3$, we have the third-order partial autocorrelation coefficient, which can be given by

$$\begin{bmatrix} I_1 \\ I_2 \\ I_3 \end{bmatrix} = \begin{bmatrix} 1 & I_1 & I_2 \\ I_1 & 1 & I_1 \\ I_2 & I_1 & 1 \end{bmatrix} \cdot \begin{bmatrix} H_1 \\ H_2 \\ H_3 \end{bmatrix}, \tag{32}$$

in which $J_3=H_3$ is the third-order partial autocorrelation coefficient. Among these matrix equations, equation (30) is a special case. It suggests that the first-order autocorrelation coefficient equals the first-order partial autocorrelation coefficient, which in turn equals the first-order auto-regression coefficient. For equations (31) and (32), we can calculate the autoregressive coefficient by means of finding the inverse matrix of the autocorrelation coefficient matrix. The last autoregressive coefficient gives the partial autocorrelation coefficient value. The others can be obtained by analogy. The spatial autocorrelation functions based on Moran's index can be generalized to those based on Geary's coefficient and Getis-Ord's index, which will be discussed after empirical analyses. For n spatial elements, the correlation number is $n*n$. thus, based on significance level of 0.05, the standard deviation of the spatial ACF and PACF can be estimated by the formula, $1/n$.

2.5 Spatial autocorrelation functions based on Geary's *C* and Getis' *G*

In order to carry out more comprehensive spatial autocorrelation analysis, the spatial autocorrelation functions may be extended to more spatial statistical measurements. Besides Moran's index, the common spatial autocorrelation measurements include Geary's coefficient and Getis-Ord's index (Geary, 1954; Getis and Ord, 1992). The former is often termed Geary's *C*, and the latter is also termed Getis's *G* for short in literature. It is easy to generalize the 2-dimensional spatial autocorrelation functions to Geary's coefficient according to the association of Moran's index with Geary's coefficient. In theory, Geary's coefficient is equivalent to Moran's index, but in practice, the former is based on sample, while the latter is based on population (Chen, 2013b; 2016). Geary's coefficient can be expressed in the following form (Geary, 1954):

$$C=\frac{(n-1)\sum_{i=1}^{n}\sum_{j=1}^{n}v_{ij}(x_i-x_j)^2}{2\sum_{i=1}^{n}\sum_{j=1}^{n}v_{ij}\sum_{i=1}^{n}(x_i-\bar{x})^2}=\frac{(n-1)\sum_{i=1}^{n}\sum_{j=1}^{n}w_{ij}(x_i-x_j)^2}{2\sum_{i=1}^{n}(x_i-\bar{x})^2}. \tag{33}$$

Based on matrix and vector, equation (33) can be simplified to the following form

$$C=\frac{n-1}{n}(e^{\mathrm{T}}Wz^2-z^{\mathrm{T}}Wz)=\frac{n-1}{n}(e^{\mathrm{T}}Wz^2-I), \tag{34}$$

where $e=[1\ 1\ \ldots\ 1]^{\mathrm{T}}$, $z^2=[z_1^2\ z_2^2\ \ldots\ z_n^2]^{\mathrm{T}}$. Equation (34) gives the exact relation between Moran's index I and Geary's coefficient C. Introducing the spatial displacement parameter into equation (33) yields two autocorrelation functions as follows

$$C(r)=\frac{n-1}{n}[e^{\mathrm{T}}W(r)z^2-I(r)]=\frac{n-1}{n}[e^{\mathrm{T}}W(r)z^2-z^{\mathrm{T}}W(r)z], \tag{35}$$

$$C^*(r)=\frac{n-1}{n}[e^{\mathrm{T}}W^*(r)z^2-I^*(r)]=\frac{n-1}{n}[e^{\mathrm{T}}W^*(r)z^2-z^{\mathrm{T}}W^*(r)z]. \tag{36}$$

Clearly, equation (35) is based on the standard unitized spatial weight matrix, corresponding to equation (23), while equation (36) is based on the quasi-unitized spatial weight matrix, corresponding to equation (27).

Further, the analytical process of spatial autocorrelation functions can be generalized to Getis' index. Based on the unitized size vector, the formula of Getis' index can be simplified to the form similar to the new expression of Moran's index, equation (5). Generally specking G[illegible]tis's index, G, is expressed as below (Getis and Ord, 1992):

$$G=\frac{\sum_{i=1}^{n}\sum_{j=1}^{n}w_{ij}x_ix_j}{\sum_{i=1}^{n}\sum_{j=1}^{n}x_ix_j}. \tag{37}$$

The notation is the same as those in equation (1). Suppose that for the outer product x_ix_j, $i=j$ can be kept, but for weight, w_{ij}, $i=j$ is rejected. Using unitized matrix and unitized vector, we can rewritten equation (37) in the following simple form (Chen, 2020)

$$G=y^{\mathrm{T}}Wy, \tag{38}$$

where $y=x/S=[y_1, y_2, \ldots, y_n]^{\mathrm{T}}$ represents the unitized vector of x. The elements of y is defined as below:

$$y_i=\frac{x_i}{S}=x_i/\sum_{i=1}^{n}x_i=\frac{x_i}{n\bar{x}}, \tag{39}$$

where the sum of x is

$$S = \sum_{i=1}^{n} x_i \, . \tag{40}$$

Now, introducing the spatial displacement parameter r into equation (37) produces two autocorrelation functions as below

$$G(r) = y^{\mathrm{T}} W(r) y \, , \tag{41}$$

$$G^{*}(r) = y^{\mathrm{T}} W^{*}(r) y \, . \tag{42}$$

Similar to equations (35) and (36), equation (41) is based on the standard unitized spatial weight matrix, corresponding to equation (23), while equation (42) is based on the quasi-unitized spatial weight matrix, corresponding to equation (27).

3 Materials and Methods

3.1 Data and analytical approach

The analytical process of spatial autocorrelation functions can be used to research the dynamic spatial structure China's system of cities. As a case demonstration of new methodology, only the capital cities of the 31 provinces, autonomous regions, and municipalities directly under the Central Government of China are taken into consideration for simplicity. The urban population is employed as a size measurement, while the distances by train between any two cities act as a spatial contiguity measurement. The census data of the urban population in 2000 and 2010 are available from the Chinese website, and the railroad distance matrix can be found in many Chinese road atlases (Chen, 2013b; Chen, 2016; Chen, 2020). The cities of Haikou and Lhasa are not taken into account in this study. Located ~~in~~ Hainan Island, Haikou is the capital of Hainan Province and ~~do not be~~ linked to other cities by railway. Lhasa is the capital of the Tibetan Autonomous Region, located on the Qinghai Tibet Plateau. Because of the plateau climate, Lhasa is loosely connected with other mainland cities of China. In fact, the Qinghai Tibet railway ~~has been being~~ under construction. Therefore, 29 Chinese capital cities are actually included in the datasets. In this case, the spatial sample size of urban population is n=29 (See Files S1 and S2). First of all, ~~use the~~ staircase function to determine a spatial contiguity matrix based on a threshold distance r. Then, the three-step method ~~can be~~ employed to calculate Moran's index and Getis-Ord's index (Chen, 2013b; Chen, 2020). The

process is as follows. Step 1: standardizing the size vector *x* yields standardized size vector *z*. Step 2: unitizing the spatial contiguity matrix based on a threshold distance *r* yields a spatial weight matrix *W*(*r*). Step 3: computing Moran's index using equation (23). The values of Moran's index can be converted into the corresponding values of Geary's coefficient with equation (34). As for Getis's index, the standardized size variable *z* should be replaced by the utilized size variable *y*, and the formula is equation (38). Changing the distance threshold *r* value yields different values of Moran's index, Geary's coefficient, and Getis's index. Thus we have different spatial autocorrelation functions. The main calculation process of spatial autocorrelation functions can be illustrated as follows (Figure 1). It is easy to realize the whole calculation process by programming the computer (See File S3).

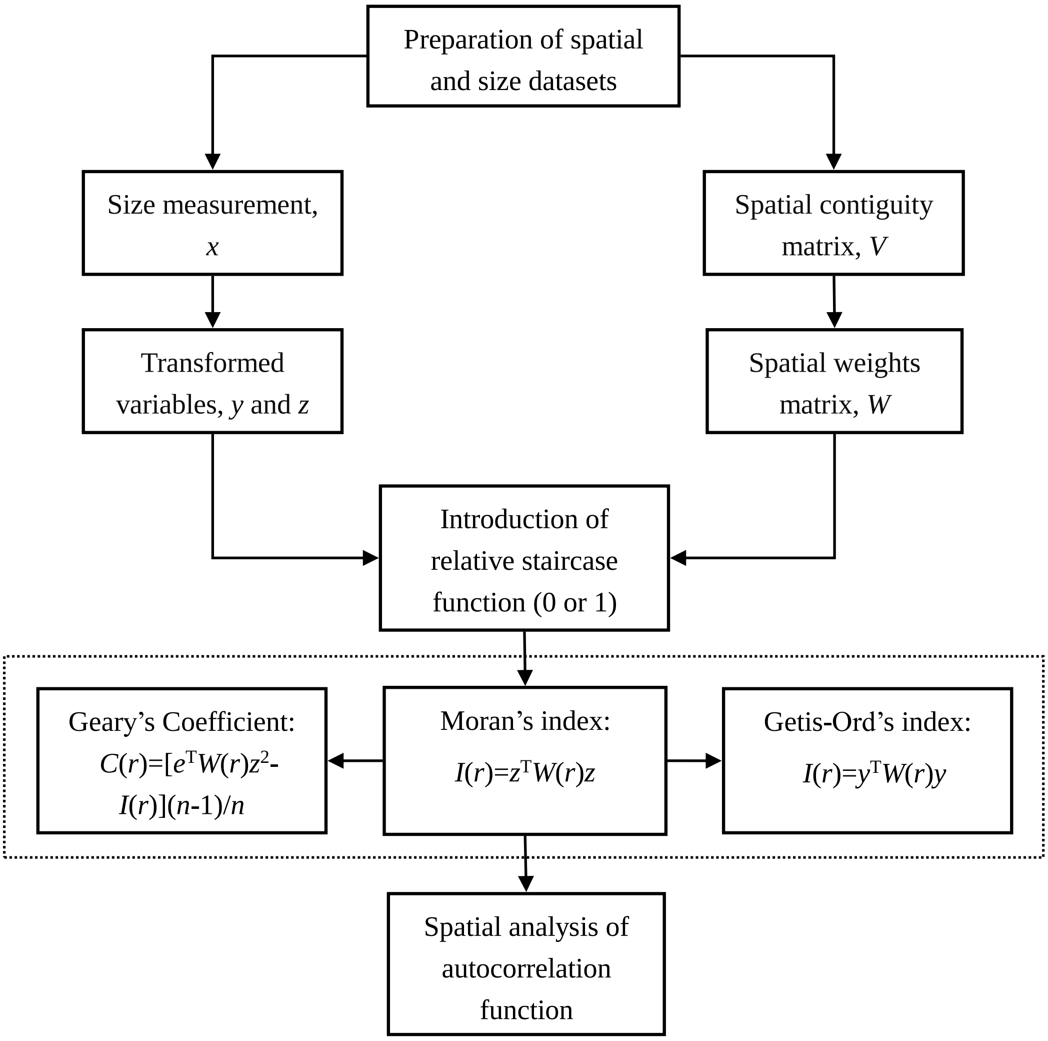

Figure 1 A flow chart of data processing, parameter estimation, and spatial autocorrelation function analyses

(**Note**: The analytical process is based on the improved analytical processes based on Moran's *I*, Geary's *C* and Getis-Ord's *G*. Based on *x*, *y* represents the unitized size variable, and *z* represents the standardized variable.)

Spatial analytical process and results rely heavily on the definition and structure of spatial contiguity matrix. Two aspects of factors in structure significantly impact on analytical process. One is diagonal elements, and the other is sum of spatial contiguity values. (1) *Diagonal elements of spatial contiguity matrix*. For conventional spatial autocorrelation analysis, the diagonal elements should be removed; while for spatial correlation dimension analysis, the diagonal elements had better be taken into account. As a matter of fact, theoretical geographers and spatial statisticians have taken into account the diagonal elements for the spatial weight matrix (Getis and Ord, 1992; Ord and Getis, 1995). Where generalized spatial autocorrelation functions are concerned, the diagonal elements of spatial contiguity matrix should not be zero. As for special fractal analysis, the diagonal element can be overlooked. (2) *The sum of spatial contiguity matrix*. For theoretical spatial autocorrelation function, the sum varies with the yardstick length. However, for practical spatial autocorrelation function, the sum of spatial contiguity matrix should be fixed to the original sum value. Different diagonal elements plus different definitions of sum of spatial contiguity matrix lead to four approaches to autocorrelation function analyses (Table 1).

Table 1 Four possible types of calculation approaches to spatial autocorrelation functions based on different diagonal elements and means of spatial contiguity matrix

	**Variable sum of distance matrix [V]**	**Fixed sum of distance matrix [F]**
**All elements (including diagonal elements) [D]**	[D+V] Generalized Moran's function, $I^*(r)$; the sum of spatial contiguity matrix elements is $N(r)$	[D+F] Generalized Moran's function, $I_f^*(r)$, the sum of spatial contiguity matrix elements is N^2
**Partial elements (excluding diagonal elements) [N]**	[N+V] Conventional Moran's function, $I(r)$; the sum of spatial contiguity matrix elements is $N(r)$-N	[N+F] Conventional Moran's function, $I_f(r)$; the sum of spatial contiguity matrix elements is $N(N$-1)
**Application direction**	Theoretical study and fractal analysis	Practical study and spatial autocorrelation analysis

3.2 Empirical analysis

Spatial contiguity matrix in spatial autocorrelation analysis bears analogy with the time lag

parameter in time series analysis. Normalizing a spatial contiguity matrix yields a spatial weight matrix, and the former is equivalent to the latter. Therefore, spatial weight matrix is always confused with spatial contiguity matrix in literature. A spatial contiguity matrix is based on generalized distance matrix and must satisfy the axiom of distance. This suggests that a spatial weight matrix must be a nonnegative definite symmetric matrix. It is easy to generate a spatial contiguity matrix by using a weight function (Chen, 2012; Getis, 2009). For *n* elements in a geographical system, a spatial contiguity matrix, $V(r)$, can be produced by means of equation (17). Normalizing the matrix V yields spatial weight matrix $W(r)$. Changing the distance threshold, i.e., the yardstick length r, results different weight matrix $W(r)$, and thus results in different Moran's index $I(r)$. A set of Moran's index values compose Moran's function. Moran's autocorrelation function can be turned into Moran's partial autocorrelation function through Yule-Walker recursive equation. The results are tabulated as below (Table 2).

Table 2 Datasets for spatial autocorrelation function (ACF) and spatial partial autocorrelation function (PACF) based on Moran's index (Partial results)

**Scale**	**2000 (Fifth census data)**				**2010 (Sixth census data)**			
r	D+F		N+F		D+F		N+F	
	ACF $I^*(r)$	PACF $J^*(r)$	ACF $I(r)$	PACF $J(r)$	ACF $I^*(r)$	PACF $J^*(r)$	ACF $I(r)$	PACF $J(r)$
**150**	0.0384	0.0384	0.0040	0.0040	0.0412	0.0412	0.0069	0.0069
**250**	0.0372	0.0357	0.0028	0.0028	0.0424	0.0408	0.0082	0.0081
**350**	0.0344	0.0318	-0.0001	-0.0001	0.0404	0.0372	0.0061	0.0060
**450**	0.0309	0.0273	-0.0037	-0.0037	0.0375	0.0329	0.0031	0.0030
**550**	0.0291	0.0248	-0.0056	-0.0056	0.0334	0.0278	-0.0011	-0.0013
**650**	0.0264	0.0216	-0.0083	-0.0083	0.0327	0.0264	-0.0018	-0.0019
**750**	0.0254	0.0202	-0.0094	-0.0093	0.0294	0.0225	-0.0052	-0.0052
**850**	0.0176	0.0120	-0.0175	-0.0174	0.0201	0.0127	-0.0149	-0.0148
**950**	0.0199	0.0145	-0.0151	-0.0150	0.0230	0.0159	-0.0119	-0.0115
**1050**	0.0109	0.0054	-0.0245	-0.0244	0.0121	0.0049	-0.0232	-0.0228
**1150**	0.0119	0.0069	-0.0234	-0.0234	0.0117	0.0052	-0.0236	-0.0229
**1250**	0.0203	0.0157	-0.0147	-0.0148	0.0143	0.0085	-0.0209	-0.0201
**1350**	0.0125	0.0076	-0.0228	-0.0231	0.0110	0.0055	-0.0243	-0.0235
**1450**	0.0122	0.0075	-0.0231	-0.0238	0.0078	0.0027	-0.0276	-0.0269
**1550**	0.0301	0.0257	-0.0045	-0.0055	0.0270	0.0227	-0.0078	-0.0071
**1650**	0.0222	0.0167	-0.0127	-0.0141	0.0214	0.0161	-0.0136	-0.0134
**1750**	0.0176	0.0115	-0.0175	-0.0194	0.0170	0.0112	-0.0181	-0.0184

**1850**	0.0255	0.0193	-0.0093	-0.0116	0.0224	0.0163	-0.0125	-0.0133
**1950**	0.0195	0.0126	-0.0155	-0.0182	0.0185	0.0119	-0.0165	-0.0177
**2050**	0.0243	0.0173	-0.0106	-0.0136	0.0224	0.0155	-0.0125	-0.0142
**2150**	0.0116	0.0040	-0.0237	-0.0272	0.0098	0.0022	-0.0256	-0.0279
**2250**	0.0029	-0.0043	-0.0327	-0.0366	0.0003	-0.0071	-0.0354	-0.0382
**2350**	0.0157	0.0097	-0.0194	-0.0235	0.0128	0.0068	-0.0225	-0.0253
**2450**	0.0034	-0.0030	-0.0322	-0.0370	0.0032	-0.0028	-0.0324	-0.0359
**2550**	0.0139	0.0087	-0.0213	-0.0264	0.0135	0.0086	-0.0217	-0.0254
**2650**	0.0078	0.0026	-0.0277	-0.0333	0.0076	0.0028	-0.0278	-0.0320
**2750**	0.0039	-0.0013	-0.0317	-0.0385	0.0025	-0.0021	-0.0331	-0.0382
**2850**	0.0019	-0.0025	-0.0338	-0.0413	0.0006	-0.0032	-0.0351	-0.0408
**2950**	0.0022	-0.0016	-0.0334	-0.0421	0.0016	-0.0015	-0.0341	-0.0408
**3050**	-0.0046	-0.0085	-0.0405	-0.0512	-0.0046	-0.0076	-0.0405	-0.0488

Note: (1) Only partial results are tabulated. More results are attached in the Supporting Information files. (2) **D** implies that diagonal elements are taken into account, **N** denotes that diagonal elements are deleted, and **F** means fixed mean values of spatial contiguity matrix elements. (3) ACF represents spatial autocorrelation function, and PACF refers to spatial partial autocorrelation function.

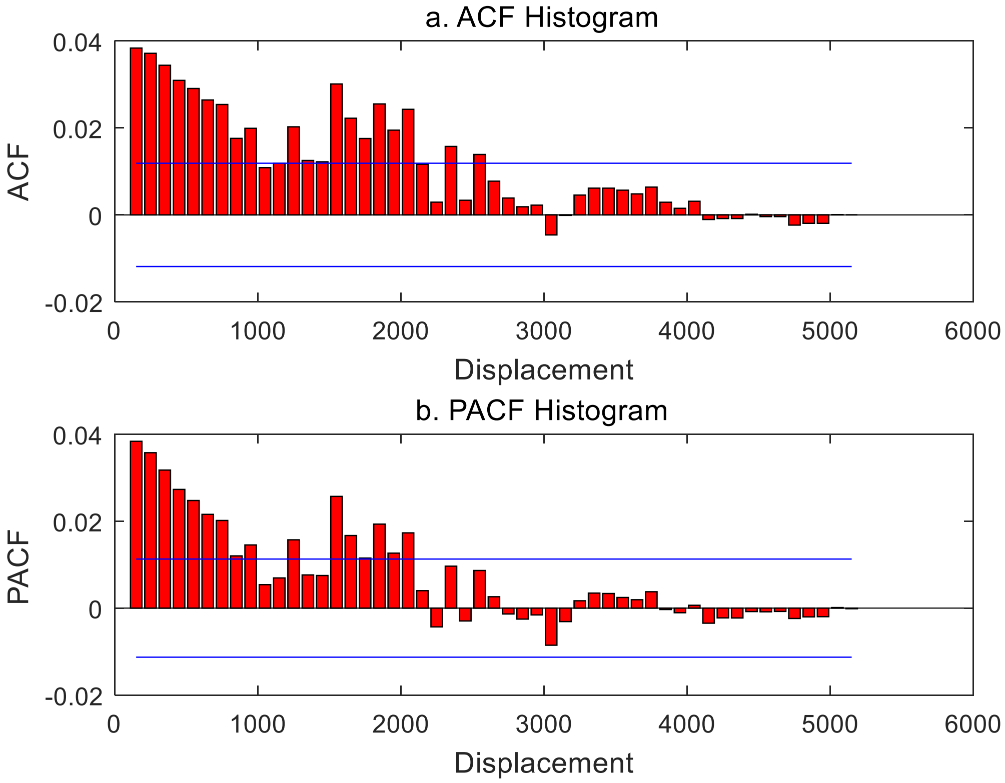

Figure 2 Spatial autocorrelation function and partial autocorrelation function of Chinese cities based on generalized Moran's index (2000)

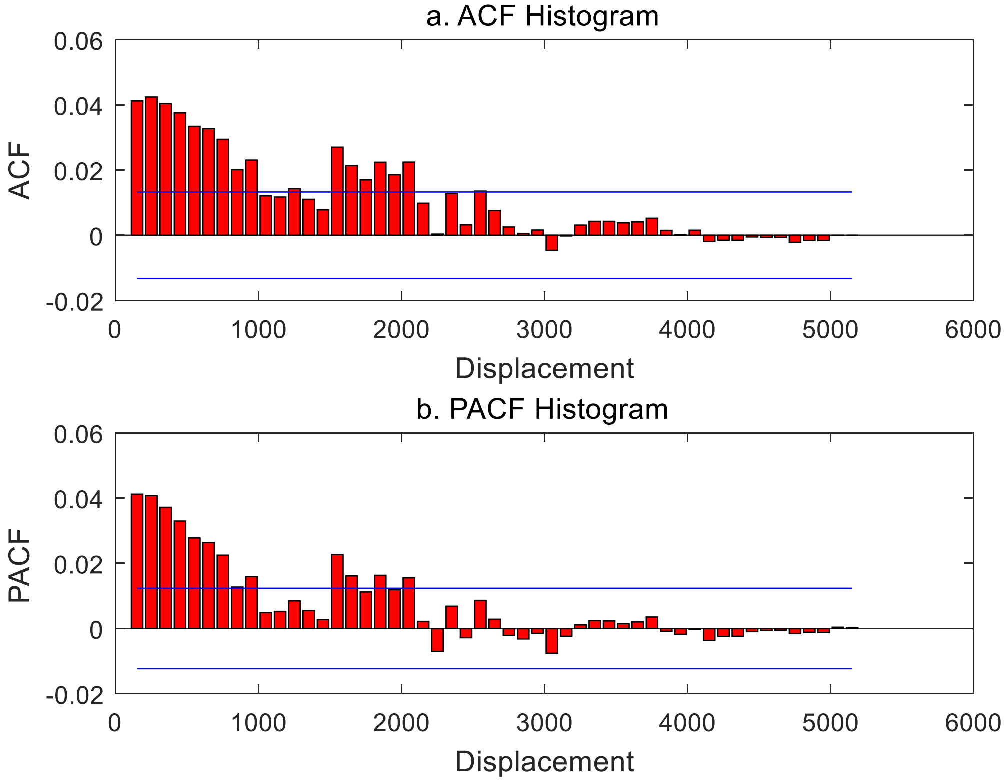

Figure 3 Spatial autocorrelation function and partial autocorrelation function of Chinese cities based on generalized Moran's index (2010)

First of all, ~~let's~~ investigate the generalized spatial autocorrelation function and the corresponding spatial partial autocorrelation function. These functions reflect the distance decay effect. The generalized autocorrelation function is based on the spatial contiguity matrix with non-zero diagonal elements, and the sum of the matrix elements is fixed to a constant n^2=29*29=841. That is to say, for every yardstick r, the number 841 is employed to divide the sum of spatial contiguity matrix elements, and the normalized results represent [illegible]atial weight matrix. This autocorrelation coefficient includes two parts: one is i correlates i and j correlates j (based on diagonal elements), and the other, i correlates j and j correlates i (based on the elements outside the diagonal of the matrix). The dynamic properties of the generalized spatial autocorrelation are as below. (1) With the increase of threshold distance, [illegible]ocorrelation function and partial autocorrelation function show wave attenuation. (2) The shape of [illegible]ocorrelation function curve is similar to that of partial autocorrelation function curve. (3) From 2000 to 2010, the shape of autocorrelation function curve ~~bear~~ [illegible] significant change (Figure 2, Figure 3). Therefore, it can be concluded that the spatial relationship between Chinese cities is relatively stable, and the direct relationship between different

cities is relatively weak. From about 2750 km to 2950 km, the positive correlation becomes weak and even turns to negative correlation. This indicates that the distance 750 to 2950 km is one of characteristic correlation scale in the spatial distribution of Chinese cities.

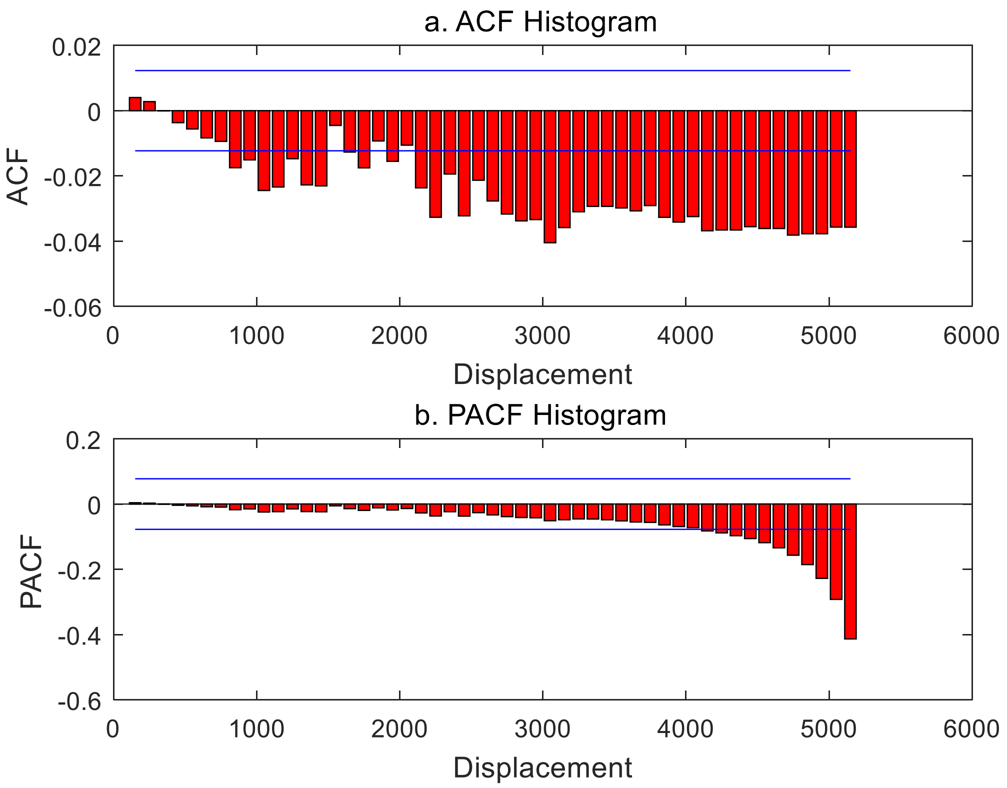

Figure 4 Spatial autocorrelation function and partial autocorrelation function of Chinese cities based on conventional Moran's index (2000)

Secondly, let's examine the standard spatial autocorrelation function and partial autocorrelation function. These functions reflect spatial transition of positive autocorrelation and negative autocorrelation. The standard autocorrelation function is based on the spatial contiguity matrix with zero diagonal elements, and the sum of the matrix elements is fixed to a constant $(n\text{-}1)n$=28*29=812 for different yardstick r. That is, the sum of spatial contiguity matrix elements is divided by the number 812, and the normalized results serve as spatial weight matrix. This autocorrelation coefficient includes only one part, namely, i correlates j and j correlates i (based on the elements outside the diagonal of the matrix). Another part, i.e., i correlates i and j correlates j (based on diagonal elements), is ignored. The dynamic properties of the standard spatial autocorrelation are as follows. (1) The short-distance positive correlation is very weak, and the long-distance negative

correlation is significant; (2) The indirect correlation reflected by autocorrelation function is significant, while the direct correlation reflected by partial autocorrelation function is relatively weak; (3) From 2000 to 2010, close range positive correlation increased, while negative correlation decreased (Figure 4, Figure 5). It can be concluded that urban spatial distribution forms heterogeneous pattern rather than agglomeration distribution. From about 250 km to 450 km, the positive correlation becomes negative. This suggests that the distance 250 to 450 km represents another characteristic correlation scale of spatial distribution of Chinese cities. Combining the results of two autocorrelation functions, two characteristic correlation scales can be obtained: one is about 250 -450 km, the other is 2750-2950 km. In fact, based on the scales ~~ranging~~ 250 to 2750, a scaling exponent, spatial correlation dimension, can be revealed from the relationships between yardstick lengths and correlation numbers of cities (this will be discussed in a companion paper).

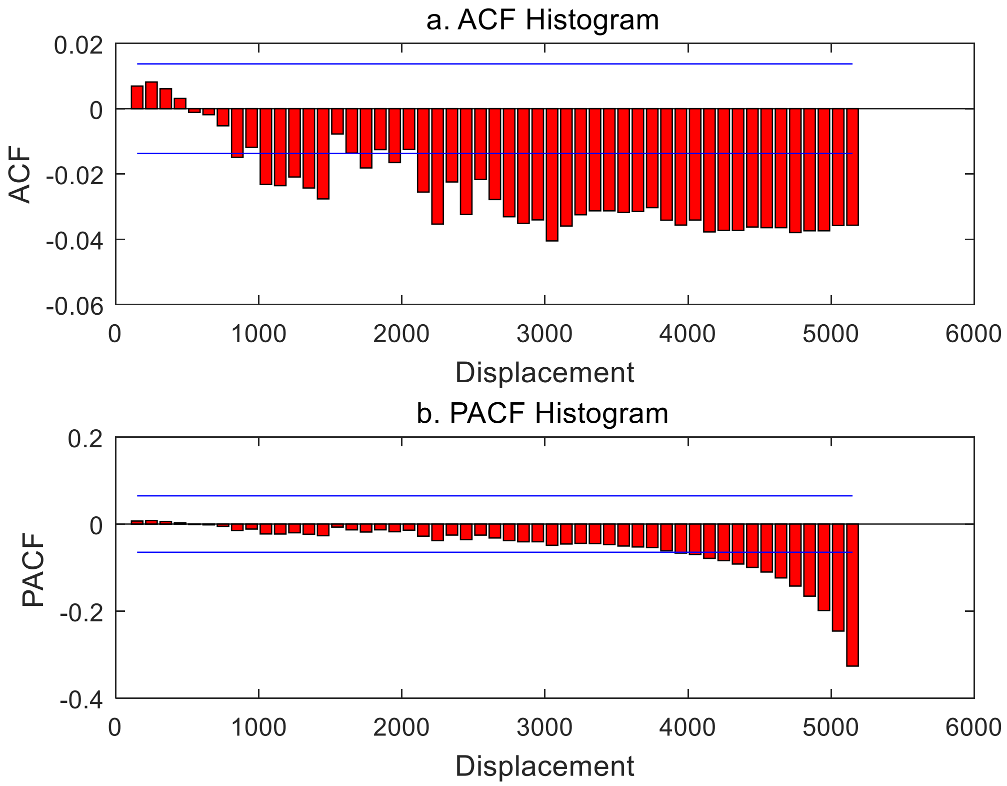

Figure 5 Spatial autocorrelation function and partial autocorrelation function of Chinese cities based on conventional Moran's index (2010)

By using spatial ACF and PACF, we can ~~real~~ the spatial autocorrelation characteristics of Chinese cities as follows. **First, the spatial autocorrelation of population among principal cities in China**

is weak. Most values of autocorrelation coefficients are less than twice the standard deviation, 1.96/29, i.e., 0.0676 (Figures 2-5). The reason lies in two aspects: one is the large territory of China, and the other is the strict registered residence management system. Therefore, on the national dimension, population migration between large cities is not easy. **Second, population flow among Chinese cities takes on self-correlation, namely, a city influence itself.** As indicated above, the self-correlation is shown by the diagonal elements (Figures 2 and 3). If the diagonal elements are taken into account, there is no significant difference between the spatial ACF and to the spatial PACF. This suggests that the diagonal elements indicative of self-correlation play a significant part in calculation of spatial ACF and PACF. **Third, the spatial autocorrelation is positive in short distance and negative in long distance.** This can be seen by standard spatial ACF and PACF (Figures 4 and 5). When the threshold distance is less than 500 km, the spatial autocorrelation coefficients are positive; while when the threshold distance is great than 1000 km, the spatial autocorrelation coefficients become negative. This suggests that similar structures are easy to form between cities in short distance, while complementary structures tend to be formed in long distance.

4 Discussion

This work is devoted to make two aspects of innovation in spatial modeling and analysis. First, in theory, the idea of spatial scaling is introduced into spatial autocorrelation modeling. The conventional spatial autocorrelation analysis is based on fixed distance threshold and characteristic scales. Moran's I is actually an eigenvalue of generalized spatial correlation matrix. In this paper, spatial autocorrelation modeling is based on variable distance threshold and scaling. Moran's I can be associated with spatial correlation dimension. Second, in methodology, the spatial correlation coefficients were generalized to spatial autocorrelation functions. Using these functions, we can make analyses of spatial dynamics of complex geographical systems.

Spatial autocorrelation analysis has gone through two stages. The first stage is reflected in biometrics. At this stage, spatial autocorrelation measurements are mainly used as auxiliary means of traditional statistical analysis. The prerequisite or basic guarantee of statistical analysis is that the sample elements are independent of each other. To measure the independence of spatial sampling results, Moran's index (Moran's I) was presented by analogy with Pearson's product-moment

correlation coefficient and autocorrelation coefficient in time series analysis (Moran, 1948; Moran, 1950). Moran's I is based on spatial populations (universes) rather than spatial samples (Chen, 2013b). As an addition, Geary's coefficient (Geary's C) was proposed by analogy with Durbin-Watson statistic (Geary, 1954), and this index is for spatial sampling analysis (Chen, 2013b). The second stage is reflected in human geography. At this stage, spatial autocorrelation becomes one of the leading tools of geospatial modeling and analysis. In the period of geographical quantitative revolution (1953-1976), ~~the~~ autocorrelation measurements were introduced into geography (Haining, 2009; Haggett *et al*, 1977). Geographers have found that few types of geospatial phenomena do not have spatial correlations, so traditional statistical analysis often fails in geographical research. Geographers changed their thinking and decided to develop a set of analytical process based on spatial autocorrelation (Cliff and Ord, 1973; Cliff and Ord, 1969; Cliff and Ord, 1981; Griffith, 2003; Odland, 1988; Wang, 2006). A number of new measurements and methods such as Getis-Ord's index (Getis's G) (Getis, 2009; Getis and Ord, 1992), local Moran's indexes and Moran's scatterplot (Anselin, 1995; Anselin, 1996), and spatial auto-regression models emerged. At the same time, spatial autocorrelation analysis continued to develop in biometrics (Sokal and Oden, 1978; Sokal and Thomson, 1987). At present, autocorrelation analysis seems to enter ~~the~~ third stage. Based on spatial autocorrelation measures and analytical processes, spatial statistics ~~has been~~ developed rapidly and applied to many areas (e.g., Beck and Sieber, 2010; Benedetti-Cecchi *et al*, 2010; Bivand *et al*, 2009; Braun *et al*, 2012; Deblauwe *et al*, 2012; Li *et al*, 2007; Tiefelsdorf, 2002; Weeks *et al*, 2004). However, if the spatial statistics ~~is~~ confined to autocorrelation coefficients and related measures, the function and uses of spatial modeling is difficult to develop further.

The above results demonstrate that the 2-dimensional spatial autocorrelation coefficients and the related statistics can be generalized to ~~the~~ 2-dimensional spatial autocorrelation functions and the related functions. A preliminary framework of spatial analysis based on autocorrelation functions ~~are~~ put forward. The main contributions of this study ~~to academy~~ can be outlined as three aspects. **First, construction of 2-dimensional spatial autocorrelation functions.** Based on Moran's index and the relative staircase function with spatial displacement parameter, two sets of spatial autocorrelation functions are constructed. **Second, definition of spatial partial autocorrelation functions.** By means of Yule-Walker recursive equation, the calculation approach of partial

autocorrelation functions are proposed. **Third, generalization of the spatial autocorrelation functions.** The 2-dimensional spatial autocorrelation function are generalized to Geary's coefficient and Getis' index and the extended autocorrelation functions are established. The main mathematical expressions can be tabulated for comparison (Table 3). The significance of developing this mathematical framework for spatial autocorrelation lies in three respects. **First, spatial information mining of geographical systems.** The spatial autocorrelation functions can be used to reveal more geographical spatial information and express more complex dynamic processes than the spatial autocorrelation coefficients. **Second, foundation of scale and scaling analysis.** If a geographical system bears characteristic scales, the spatial autocorrelation functions can be used to bring to light the characteristic length; if a geographical system has no characteristic scale, the spatial autocorrelation functions can be employed to make scaling analysis. **Third, future development of spectral analysis.** Autocorrelation functions and power/wave spectral density represents two different sides of the same coi[illegible]ased on the spatial autocorrelation functions, the method of 2-dimensional spectral analysis can be developed for geographical research in the future.

Table 3 Collections of two types of spatial autocorrelation functions and the extended results

**Type**	**Base**	**Standard SACF (*i*≠*j*)**	**Generalized SACF (*i*=*j*)**
**Basic functions**	Moran's *I*: SACF	$I(r)=z^{\mathrm{T}}W(r)z$	$I^*(r)=z^{\mathrm{T}}W^*(r)z$
	SPACF	$J(r)=f(I(r))$	$J^*(r)=f(I^*(r))$
**Extended functions**	Geary's *C*	$C(r)=\frac{n-1}{n}[e^{\mathrm{T}}W(r)z^2-I(r)]$	$C^*(r)=\frac{n-1}{n}[e^{\mathrm{T}}W^*(r)z^2-I^*(r)]$
	Getis's *G*	$G(r)=y^{\mathrm{T}}W(r)y$	$G^*(r)=y^{\mathrm{T}}W^*(r)y$
**Difference**	SWM	$W(r)=V(r)/V_0(r)$	$W^*(r)=V(r)/(n(n-1))$

The spatial weight function is based on relative staircase function. Sta[illegible]rcase function can be regarded as a special case of ne[illegible]ative exponential function. The exponential function has been employed to make sp[illegible]tial autocorrelation analysis (Chen, 2013b; Chen, 2016). It can be expressed as below

$$v_{ij} = f(r) = \exp(-\frac{d_{ij}}{\bar{d}}), \tag{43}$$

where d bar denotes the mean value of distance d_{ij}. Substituting the average distance in equation (43) with a threshold distance r yields

$$v_{ij} = f(r) = \exp(-\frac{d_{ij}}{r}), \tag{44}$$

where r refers to the threshold value of distance d_{ij}. In theory, the r value varies from 0 to infinity (∞), and the contiguity value ranges from 0 to 1. That is, we have

$$v_{ij} = f(r) = \exp(-\frac{d_{ij}}{r}) = \begin{cases} 1, & r \to \infty \\ 0, & r \to 0 \end{cases}, \tag{45}$$

which suggests that the v_{ij} values are normalized values. The staircase function gives a set of discrete values of spatial weight, while the exponential function gives a set of continuous values of spatial values. The question is when to use discrete weights and when to use continuous weights. This problem remains to be solved in future theoretical and empirical studies.

The new development of a theory or a method always gives rise to a series of new problems. New problems will lead to further exploration about the theory or the method. The main shortcomings of this work are as follows. **First, the local spatial autocorrelation functions have not been taken into consideration.** Moran's index, Geary's coefficient, and Getis' index can be used to measure local spatial autocorrelation. However, local spatial coefficients have not been generalized to local spatial autocorrelation functions. **Second, the auto-regression models have not been built.** Autocorrelation and auto-regression represent two different sides of the same coin. How ~~to make~~ the auto-regression models, which can give the partial autocorrelation coefficients? This is a pending question. **Third, ~~the~~ scaling analysis has not been introduced into spatial autocorrelation functions.** In principle, spatial autocorrelation functions can be associated with spatial correlation dimension. However, the strict mathematical relationships between spatial correlation dimension and spatial autocorrelation functions remain to be demonstrated. Limited to the length of the article, the above questions will be reported in subsequent research papers.

5 Conclusions

A new analytical framework ~~of~~ based on a series of spatial autocorrelation functions have been demonstrated with mathematical derivation. A case study is presented to show how to make use of this analytical process. Next ~~step,~~ we ~~can~~ further develop the related spatial analytical methods based on spatial autocorrelation functions, including spatial cross-correlation functions, spatial auto-regression modeling, spatial wave-spectral analysis. The main points ~~of this work~~ can be summarized as follows. **First, new spatial analytical process can be developed by spatial autocorrelation functions based on the relation staircase function.** Introducing spatial displacement into spatial weight functions, we can transform the spatial autocorrelation coefficients such as Moran's index into spatial autocorrelation functions. An autocorrelation function is a set comprising a series of autocorrelation coefficients. A spatial autocorrelation coefficient can be used to characterize the simple spatial correlation and structure, while a spatial autocorrelation function can be employed to describe the complex spatial correlation and dynamics. **Second, spatial partial autocorrelation functions can be used to assist spatial autocorrelation function analysis.** Using Yule-Walker recursive equation, we can convert the spatial autocorrelation function based on Moran's index into spatial partial autocorrelation functions. Spatial autocorrelation functions reflect both direct and indirect spatial autocorrelation processes in a system, while spatial partial autocorrelation functions can be employed to display the pure direct autocorrelation process. **Third, the spatial autocorrelation functions can be extended by means of more spatial autocorrelation measurements.** The spatial autocorrelation functions can be generalized to the autocorrelation functions based on Geary's coefficient and Getis' index. Different autocorrelation functions have different uses in spatial analysis. Using the spatial autocorrelation functions, we can mine more geographical spatial information, seek the characteristic scales for spatial modeling and quantitative analysis, or reveal the hidden scaling in complex geographical patterns and processes.

Acknowledgement:

This research was sponsored by the National Natural Science Foundation of China (Grant No. 41671167. See: http://isisn.nsfc.gov.cn/egrantweb/). The support is gratefully acknowledged.

Supporting Information

File S1. Datasets of urban population and railway distances in 2000 for calculating spatial autocorrelation and partial autocorrelation functions. This file contains the original or preliminarily processed data of 2000 used in this paper. It provides two complete processes of computing spatial autocorrelation function (ACF) and partial autocorrelation function (PACF). (XLSX)

File S2. Datasets of urban population and railway distances in 2010 for calculating spatial autocorrelation and partial autocorrelation functions. This file contains the original or preliminarily processed data of 2010 used in this paper. (XLSX)

File S3. Four Matlab programs for spatial autocorrelation and partial autocorrelation function analysis based on Moran's index. It provides four Matlab programs for calculating spatial autocorrelation function (ACF) and partial autocorrelation function (PACF). Among the four programs, two is significant: one is based on diagonal elements and variable weights, and the other is based on zero diagonal elements and fixed weights. Readers can employ the programs to carry out spatial ACF and PACF analyses by substituting author's data with their own data. (MAT)

References

Anselin L (1995). Local indicators of spatial association—LISA. *Geographical Analysis*, 27(2): 93-115

Anselin L (1996). The Moran scatterplot as an ESDA tool to assess local instability in spatial association. In: M. Fischer, H.J. Scholten, D. Unwin (Eds). *Spatial Analytical Perspectives on GIS*. London: Taylor & Francis, pp111-125

Beck J, Sieber A (2010). Is the spatial distribution of mankind's most basic economic traits determined by climate and soil alone? *PLoS ONE*, 5(5): e10416

Benedetti-Cecchi L, Iken K, Konar B, Cruz-Motta J, Knowlton A, *et al*. (2010). Spatial relationships between polychaete assemblages and environmental variables over broad geographical scales. *PLoS ONE*, 5(9): e12946

Bivand RS, Müller W, Reder M (2009). Power calculations for global and local Moran's *I*. *Computational Statistics and Data Analysis*, 53: 2859-2872

Bowerman BL, O'Connell RT (1993). *Forecasting and Time Series: An Applied Approach (Third Edition)*. Pacific Grove, CA: Thomson Brooks

Box GEP, Jenkins GM, Reinsel GC (1994). *Time Series Analysis Forecasting and Control (Third Edition)*. Upper Saddle River, NJ: Prentice Hall

Braun A, Auerswald K, Geist J (2012). Drivers and spatio-temporal extent of hyporheic patch variation: Implications for sampling. *PLoS ONE*, 7(7): e42046

Chen YG (2012). On the four types of weight functions for spatial contiguity matrix. *Letters in Spatial and Resource Sciences*, 5(2): 65-72

Chen YG (2013a). Fractal analytical approach of urban form based on spatial correlation function. *Chaos, Solitons & Fractals*, 49(1): 47-60

Chen YG (2013b). New approaches for calculating Moran's index of spatial autocorrelation. *PLoS ONE*, 8(7): e68336

Chen YG (2015). A new methodology of spatial cross-correlation analysis. *PLoS ONE*, 10(5): e0126158

Chen YG (2016). Spatial autocorrelation approaches to testing residuals from least squares regression. *PLoS ONE*, 11(1): e0146865

Chen YG (2020). New framework of Getis-Ord's indexes associating spatial autocorrelation with interaction. *PLoS ONE*, 15(7): e0236765

Cliff AD, Ord JK (1969). The problem of spatial autocorrelation. In: A.J. Scott (Ed.). *London Papers in Regional Science 1, Studies in Regional Science*. London: Poin, pp25-55

Cliff AD, Ord JK (1973). *Spatial Autocorrelation*. London: Pion

Cliff AD, Ord JK (1981). *Spatial Processes: Models and Applications*. London: Pion

Deblauwe V, Kennel P, Couteron P (2012). Testing pairwise association between spatially autocorrelated variables: A new approach using surrogate lattice data. *PLoS ONE*, 7(11): e48766

Geary RC (1954). The contiguity ratio and statistical mapping. *The Incorporated Statistician*, 5(3): 115–145

Getis A (2009). Spatial weights matrices. *Geographical Analysis*, 41 (4): 404–410

Getis A, Ord JK (1992). The analysis of spatial association by use of distance statistic. *Geographical Analysis*, 24(3):189-206

Griffith DA (2003). *Spatial Autocorrelation and Spatial Filtering: Gaining Understanding Through Theory and Scientific Visualization*. New York: Springer

Haggett P, Cliff AD, Frey A (1977). *Locational Analysis in Human Geography*. London: Edward Arnold

Haining RP (2009). Spatial autocorrelation and the quantitative revolution. *Geographical Analysis*, 41 (4): 364-374

Hao BL (1986). Fractals and fractal dimensions. *Science*, 38 (1): 9-17 (in Chinese)

Li H, Calder CA, Cressie N (2007). Beyond Moran's *I*: Testing for spatial dependence based on the spatial autoregressive model. *Geographical Analysis*, 39(4): 357-375

Moran PAP (1948). The interpretation of statistical maps. *Journal of the Royal Statistical Society, Series B*, 37(2): 243-251

Moran PAP (1950). Notes on continuous stochastic phenomena. *Biometrika*, 37(1-2): 17-33

Odland J (1988). *Spatial Autocorrelation*. London: SAGE Publications

Ord JK, Getis A (1995). Local spatial autocorrelation statistics: distributional issues and an application. *Geographical Analysis*, 27(4): 286-306

Sokal RR, Oden NL (1978). Spatial autocorrelation in biology. 1. Methodology. *Biological Journal of the Linnean Society*, 10(2): 199-228

Sokal RR, Thomson JD (1987). Applications of spatial autocorrelation in ecology. In: P. Legendre, L. Legendre (Eds). *Developments in Numerical Ecology, NATO ASI Series, Vol. G14*. Berlin: Springer-Verlag, pp431-466

Tiefelsdorf M (2002). The saddle point approximation of Moran's I and local Moran's I_i reference distributions and their numerical evaluation. *Geographical Analysis*, 34(3): 187-206

Wang JF (2006). *Spatial Analysis*. Beijing: Scientific Press [In Chinese]

Weeks JR, Getis A, Hill AG, Gadalla MS, Rashed T (2004). The fertility transition in Eqypt: Intraurban patterns in Cairo. *Annals of the Association of American Geographers*, 94(1): 74-93

Click here to access/download

Supporting Information - Compressed/ZIP File Archive

Supporting Information files.zip
