# Supplementary material for: An analytical process of spatial autocorrelation functions based on Moran’s index
Source: PLoS One. 2021 Apr 14;16(4):e0249589. doi: 10.1371/journal.pone.0249589 (PMC8046215; doi:10.1371/journal.pone.0249589)
Supplement: Attachment — Submitted filename: review.pdf [file pone.0249589.s005.pdf]

Review of

An Analytical Process of Spatial Autocorrelation Functions Based on Moran's Index by Yanguang Chen

Stéphane DRAY

I have read the paper and I must admit that I am very skeptical about the novelty of the paper. I work mainly on spatial analysis in ecology and geography. I am perhaps wrong but for me, the author proposes a revisit of very simple approaches developped in geography. He uses very technical and complicated terms (from other fields) to question the utility to develop autocorrelation functions (varying distances) to describe spatial patterns. For me these methods are correlograms (Mantel or Moran's in the sense of Legendre and Legendre (1998)) and variograms (which can be seen as an extension of Geary's C). They have been devolopped many years ago and describe spatial patterns with various lags. Perhaps I can miss something but I do not see any difference to what is proposed here (except the partial aspect). I can miss something (as the text is very complicated and hard to read, it can be possible) but as the author do not cite these well known and very similar tools, it is hard to have an idea of the novelty of this contribution.

It seems that the author ignore a lot of published works (see above). For instance, a lot of papers works on eigenvalues/vectors of spatial weighting matrices (e.g., de Jong et al., 1984; Dray, 2011; Dray et al., 2006; Griffith, 1996, 2003) are not cited while the author suggest very similar future developments (third point in bold p. 22). Ignoring all the relevant previous works on this question do not help to see really if the author proposed something new (for me, only the partial indexes.. but I am not sure as I do not search for any relevant publications on the subject in spatial context) or rediscover traditional tools with another (complicated) viewpoint.

To be honest, I do not send comments on the whole manuscript as I think that all the major comments addressed here should be considered to envisage if the paper makes sense or not. The author should drastically modify this paper to better integrate his viewpoint and the associated proposed approach with the existing literature. Hence, this is a sample of comments (not an exhaustive list):

- p. 2, L. 6-10: I do not know what are *characteristics scales.* Please

explain, give references, say why it is useful and/or important and explain what is the concrete problem associated.

- p. 2, L. 12-23: I do not agree with this view and I am not convinced by the statements of the author. First, the text is quite hard to follow. Please give examples of statistics/approaches with appropriate referecnces, and be clearer on the different methods described. For me, the spatio-temporal analogy is as follows: (i) correlations, temporal autocorrelation (von Neumann, 1941; von Neumann et al., 1941) and spatial autocorrelation (Geary, 1954; Moran, 1950) and (ii) autocorrelation functions (ACF, PACF) and correlograms, variograms. The first approaches looks at (dis)similarities between two values that can be separated by a lag (in time or space) while the second look at the same patterns but provides a functional description at different lag values. The spatial $n \times n$ weighting matrix (where n corresponds to the number of sampling units) corresponds to a matrix with the same dimensions that define that one observation is the neighbor (possibly weigted) of its precedent and subsequent observations (in a symmetric definition of neighboring, only subsequent for an asymmetric definition). As this part motivates the work, this comment should be carefully considered.

- p. 4, L. last: The discussion about normalization is useless. I think that the author should focus on one subject and remove these parts. Moreover, the author states that total normalization is often used but row-standardization (or normalization) is much more used in geography or ecology as it simplifies the interpretation (Moran's scatterplot Anselin, 1996)

- p. 5: I do not understand what you want to explain here. Why not directly give the formula of eigenvalues/eigenvectors. Why do you need all this mathematical playing of matrix products?

- there are a lot of statements in the paper that are more opininions than reality (e.g., "A good model is ..." (P. 6, L. 2). Please avoid this type of sentences.

- bibliography is really centred on some publications (except in the discussion) but a lot of very traditional/approaches works are ignored and not cited (correlograms, row standardization of the spatial weighting matrix) during the methodological description of methods

PS: Adding line numbering would be appreciated to facilitate the reviewing process

Cheers

References

Anselin, L. (1996). The Moran scatterplot as an ESDA tool to assess local instability in spatial association. In Fischer, M. M., Scholten, H. J., and Unwin, D., editors, *Spatial analytical perspectives on GIS*, pages 111–125. Taylor and Francis, London.

de Jong, P., Sprenger, C., and van Veen, F. (1984). On extreme values of Moran's I and Geary's C. *Geographical Analysis*, 16(1):17–24.

Dray, S. (2011). A new perspective about Moran's coefficient: spatial autocorrelation as a linear regression problem. *Geographical Analysis*, 43:127–141.

Dray, S., Legendre, P., and Peres-Neto, P. R. (2006). Spatial modeling: a comprehensive framework for principal coordinate analysis of neighbor matrices (PCNM). *Ecological Modelling*, 196:483–493.

Geary, R. C. (1954). The contiguity ratio and statistical mapping. *The Incorporated Statistician*, 5(3):115–145.

Griffith, D. A. (1996). Spatial autocorrelation and eigenfunctions of the geographic weights matrix accompanying geo-referenced data. *Canadian Geographer*, 40(4):351–367.

Griffith, D. A. (2003). *Spatial autocorrelation and spatial filtering: gaining understanding through theory and scientific visualization*. Springer-Verlag, Berlin.

Legendre, P. and Legendre, L. (1998). *Numerical Ecology*. Elsevier Science, Amsterdam, second edition.

Moran, P. A. P. (1950). Notes on continuous stochastic phenomena. *Biometrika*, 37:17–23.

von Neumann, J. (1941). Distribution of the ratio of the mean square successive difference to the variance. *The Annals of Mathematical Statistics*, 12:367–395.

von Neumann, J., Kent, R. H., Bellinson, H. R., and Hart, B. I. (1941). The mean square successive difference. *The Annals of Mathematical Statistics*, 12:153–162.
