# Supplementary material for: An analytical process of spatial autocorrelation functions based on Moran’s index
Source: PLoS One. 2021 Apr 14;16(4):e0249589. doi: 10.1371/journal.pone.0249589 (PMC8046215; doi:10.1371/journal.pone.0249589)
Supplement: Attachment — Submitted filename: Response to Reviewers2021-01-18.docx [file pone.0249589.s006.docx]

## Response to Reviewers

### Reviewer #1

**[Reviewer #1]** This is an interesting study that attempts to unite the commonly used metrics of spatial autocorrelation such as Moran's *I* and Gettis *G* by developing matrix algebra that unites the formulae for the metrics and that supports multiscale analysis. The case study is population in major Chinese cities, and it illustrates the approach well.

**[Reply]** Thank you.

**[Reviewer #1]** I feel that the claim to link spectral power analysis with spatial analysis is not new, and that a broader literature search could reveal more prior work. Also, the paper needs a thorough proof edit of the English, as it lacks definite articles, does not balance verb tenses, mixes singulars and plurals etc. I will upload a marked up PDF that I hope the authors can use to improve the manuscript.

**[Reply]** Thank you very much for help me edit the manuscript.

### Reviewer #2

Review of *An Analytical Process of Spatial Autocorrelation Functions Based on Moran's Index* by Yanguang Chen ---Stéphane DRAY

Dear Stéphane,

I'm glad to have the opportunity to make an academic exchange with you on spatial analysis. Thank you very for your interesting and constructive comments and kind advices. The references provided by you are helpful for me to improve this paper. The work in this paper proceeded from the measurement of spatial correlation dimension, a type of fractal dimension.

I didn't study spatial autocorrelation 15 years ago. For many years, I have been studying fractal cities and systems of cities. In 1998, I designed a method to measure spatial correlation dimension of urban systems and transport networks in China (Chen and Luo, 1998). In 2001, I began to teach Mathematical Methods for Geography for graduate students in Peking University. The teaching contents include time series analysis (autocorrelation analysis, auto-regressive process, moving average process, auto-regressive moving average process, power spectral analysis). In 2006, I began to teach Quantitative Geography for undergraduates in Peking University. During the processing of teach Quantitative Geography, I found that the variable distances of measuring spatial correlation dimension could be further employed to do two things: one is to develop 2-dimensional spatial autocorrelation function analysis, and the other is to reveal the relationships between fractal dimension and Moran’s index from the prospective of scaling analysis.

Thus, in 2018, I wrote two companion papers entitled “An analytical process of spatial autocorrelation functions based on Moran's index” (this paper) and “Derivation of correlation dimension from spatial autocorrelation functions” (another paper). The preprints of these paper are released at arXiv (<https://arxiv.org/abs/1901.03837>).

In this paper, I developed spatial autocorrelation functions based on Moran’s *I*, Geary’s *C*, and Getis-Ord’s *G*. Thank you for telling me Dr. Legendre and Legendre’s work. My spatial autocorrelation functions differ from the structure function proposed by Dr. Legendre and Legendre. In short, Legendre’s works and so on are based on characteristic scale concept, and my studies are based on the idea from scaling analysis. I will explain it to you next.

- Chen YG (2019). Derivation of correlation dimension from spatial autocorrelation functions. *arXiv:1901.03837* [physics.soc-ph], https://arxiv.org/abs/1901.03837
- Chen YG (2019-2020). An analytical process of spatial autocorrelation functions based on Moran's index. *arXiv:2001.06750*, 2020 [physics.soc-ph]
- Chen YG, Luo J (1998). The fractal features of the transport network of Henan Province. *Journal of Xinyang Teachers College (Natural Science Edition)*, 11(2): 172-177 [In Chinese]

**[Reviewer #2]** I have read the paper and I must admit that I am very skeptical about the novelty of the paper. I work mainly on spatial analysis in ecology and geography. I am perhaps wrong but for me, the author proposes a revisit of very simple approaches developped in geography. He uses very technical and complicated terms (from other fields) to question the utility to develop autocorrelation functions (varying distances) to describe spatial patterns. For me these methods are correlograms (Mantel or Moran's in the sense of Legendre and Legendre (1998)) and variograms (which can be seen as an extension of Geary's *C*). They have been devolopped many years ago and describe spatial patterns with various lags. Perhaps I can miss something but I do not see any difference to what is proposed here (except the partial aspect). I can miss something (as the text is very complicated and hard to read, it can be possible) but as the author do not cite these well known and very similar tools, it is hard to have an idea of the novelty of this contribution.

**[Reply]** There are significant differences between my work and that of Legendre and Legendre (1998). It can be said that the analytical process I proposed in this paper has few essential points in common with the analysis method proposed by Legendre and Legendre (1998), except the variable distance and correlograms.

Now, to explain the similarities and differences between the structure function of Legendre and the autocorrelation functions presented in this paper, a paragraph and a table have been added to the Discussion as follows:

Spatial autocorrelation methods open new ways of geographical statistical analysis under the conditions of existing inherent correlation among spatial sampling points. In particular, it laid the foundation for spatial autoregressive modeling. However, more and more evidences showed that the measurement values of spatial autocorrelation indexes depend on size, shape, and spatial scales of geographical systems (Bjørnstad and Falck, 2001; Getis and Ord, 1992; Legendre and Legendre, 1998; Odland, 1988). At least two approaches can be used to solve this problem. One is to make spatial scaling analysis based on spatial autocorrelation indexes, and the other is develop spatial autocorrelation function analysis. One basic method of developing spatial autocorrelation functions is to make use of variable distance. Based on the variable distance defined in spatial contiguity matrices, spatial correlation function, structure function, spatial correlogram, spline correlogram, and so on, have been introduced into spatial autocorrelation processes (Bjørnstad and Falck, 2001; Legendre and Legendre, 1998; Odland, 1988). Spatial correlogram is just a result from analogy with the correlation function histogram in time series analysis. Among various methods of spatial analyses based on variable distance, the structure function advanced by Legendre and Legendre (1998) looks like the spatial autocorrelation function developed in this work. However, there is essential difference between structure function and autocorrelation function. A comparison can be drawn by tabulating the similarities and differences between structure function and spatial autocorrelation function (Table 3). In short, the structure function is based on the idea of characteristic scales, while the spatial autocorrelation function is associated with scaling analysis for geographical systems. In fact, the variable distance can be employed to find the characteristic scale of spatial autocorrelation processes (Odland, 1988).

**Table 3 The differences and similarities between structure function and spatial autocorrelation function**

| Item | Legendres’ work | Work in this paper |
| --- | --- | --- |
| Objective | Finding typical autocorrelation index | Find spatial scaling and relations to fractal dimension |
| Basic postulate | Characteristic scale | Scaling invariance |
| Statistic hypothesis | Gaussian distribution | Pareto distribution |
| Spatial contiguity definition | Kronecker's delta | Heaviside function (step function) |
| Distance conversion | Metric variable → Rank variable → Categorical variable | Metric variable → Categorical variable |
| Spatial weight matrix | (1) Based on variable mean; (2) Diagonals are zeros | (1) Based on fixed mean; (2) Diagonals are zeros or ones |
| Spatial correlation | Correlation density | Correlation cumulation |
| Measurement method | Variable distance | Variance distance |
| Measurement result | Autocorrelation coefficients | Autocorrelation and partial autocorrelation coefficients |
| Modeling result | Structure function | Autocorrelation function partial autocorrelation function |
| Representation way | Correlogram | Correlogram |
| Function | Spatial structure analysis based on characteristic scale | Spatial dynamics analysis based on scaling idea |

**[Reviewer #2]** It seems that the author ignore a lot of published works (see above). For instance, a lot of papers works on eigenvalues/vectors of spatial weighting matrices (e.g., de Jong et al., 1984; Dray, 2011; Dray et al., 2006; Griffith, 1996, 2003) are not cited while the author suggest very similar future developments (third point in bold p. 22). Ignoring all the relevant previous works on this question do not help to see really if the author proposed something new (for me, only the partial indexes.. but I am not sure as I do not search for any relevant publications on the subject in spatial context) or rediscover traditional tools with another (complicated) viewpoint.

**[Reply]** All these papers has been added to the list of references.

There are Fourier cosine transformation relationships between (auto)correlation function and energy spectrum density (Chen, 2011). In fact, I have developed the spatial analysis method of spectral analysis based on 1-dimension spatial autocorrelation function (Chen, 2013; Chen, 2019; Chen and Long, 2020). By means of Fourier transform, it is very easy to develop 2-dimensional space spectrum analysis based on the autocorrelation functions proposed in this paper.

- Chen YG (2013). Fractal analytical approach of urban form based on spatial correlation function. *Chaos, Solitons & Fractals*, 49(1): 47-60
- Chen YG (2019). Fractal dimension analysis of urban morphology based on spatial correlation functions. In: D'Acci L(ed.). *Mathematics of Urban Morphology*. Birkhäuser: Springer Nature Switzerland AG, pp21-53
- Chen YG, Long YQ (2021). Spatial signal analysis based on wave-spectral fractal scaling: A case of urban street networks. *Applied Sciences*, 11(1): 87
- Chen YG (2011). *Mathematical Methods for Geography*. Beijing: Science Press [In Chinese]

**[Reviewer #2]** To be honest, I do not send comments on the whole manuscript as I think that all the major comments addressed here should be considered to envisage if the paper makes sense or not. The author should drastically modify this paper to better integrate his viewpoint and the associated proposed approach with the existing literature. Hence, this is a sample of comments (not an exhaustive list):

**[Reply]** I have revised the paper thoroughly by referring to the comments from three reviewers.

•**[Reviewer #2]** p. 2, L. 6-10: I do not know what are characteristics scales. Please explain, give references, say why it is useful and/or important and explain what is the concrete problem associated.

**[Reply]** I explained the concept of characteristic scale in the manuscript you reviewed. However, because you did not read the whole manuscript (you said “To be honest, I do not send comments on the whole manuscript as I think that all the major comments addressed here should be considered to envisage if the paper makes sense or not”) , the explanation was overlooked by you. Now, the paragraph about characteristic scale has been revised as follows:

“Scientific description always relies heavily on a characteristic scale of a geographical system. A characteristic scale is a typical scale which can be represented by a 1-dimensional measure. Thus, characteristic scales are usually termed *characteristic length* in the literature (Feder, 1988; Hao, 2004; Liu and Liu, 1993; Takayasu, 1990; Wang and Li, 1996). In mathematics, characteristic scales include determinate radius, side length, eigenvalue, average values, and standard deviation. An eigenvalue, if it does not depend on measurement scale, can be treated as a characteristic length. Therefore, eigenvalues and eigenvectors are important in spatial autocorrelation analyses (de Jong *et al*, 1984; Dray, 2011; Dray *et al*, 2006; Griffith, 1996; Griffith, 2003).”

“Moran’s *I* is actually an eigenvalue of the generalized spatial correlation matrix. If and only if an eigenvalue bear no scale dependence, it can serve a characteristic length in spatial analysis. Unfortunately, in many cases, Moran’s *I* depends on spatial measurement scale.”

As we know, scientific research includes two processes: one is description, and the other is understanding. Science should proceed first by describing how a system and its parts work and then by understanding why (Gordon, 2005; Henry, 2002). The precise description relies heavily on mathematics and measurements (Henry, 2002). Conventional mathematical modeling and quantitative analysis are based on characteristic scales (Feder, 1988; Hao, 2004; Liu and Liu, 1993; Takayasu, 1990; Wang and Li, 1996). Unfortunately, complex systems such as cities bear no characteristic scale. In this case, the concept of characteristic scale should be replaced by the idea of scaling. Fractal geometry provides a powerful tool for describing scale-free phenomena and can be used to make scaling analysis of geographical systems.

The characteristic scale is always termed characteristic length because it is a 1-dimension measure such as radius, sidelength, mean, eigenvalue, and so on. Many scholars unconsciously use the concept of characteristic scale. Only the scientists who explore complexity and pay attention on scaling analysis emphasize the difference between characteristic length and scaling.

(1) For a geographical geometric object, if it bear determinate length, area, or volume, it has characteristic length, which can be expressed with a 1-dimensional measure such as radius and sidelenth. Otherwise, it has no characteristic length and should be performed analysis by scaling idea. (2) For a spatial sample, if its mean is determinate, the mean can be treated as a characteristic length. On the contrary, if the mean depends on sample size or measurement scale, it cannot be regarded as a characteristic length. In this case, the analytic method based on characteristic scale should be replaced by the idea from scaling law. (3) For a square matrix such covariance matrix, if its eigenvalues are determinate, the eigenvalues can be treated as characteristic lengths. On the contrary, if the eigenvalues are not determinate, or depend on measurement scale, it cannot be regarded as characteristic lengths. In this instance, the analytic method based on characteristic scale should also be taken place by the notion from scaling analysis.

Moran’s index proved to an eigenvalue of generalized spatial correlation matrix. If Moran’s index is determinate, it can be treated as a characteristic length of spatial analysis, or else, scaling idea should be introduced into spatial autocorrelation analysis.

**Table A1 Mathematical methods fall into two categories**

| Type | Basis | Method | Scope of application | Note |
| --- | --- | --- | --- | --- |
| Conventional method | Characteristic scale (Characteristic length) | Calculus, linear algebra, probability theory and statistics, etc. | Simple systems or simple aspects of complex systems | Simple systems have no complex aspect |
| Nonconventional method | Scaling (dilation symmetry) | Fractal geometry, complex network theory, wavelet analysis, renormalization group, etc. | Complex systems or complex aspects of complex systems | Complex systems may have simple aspects |

**Table A2 Geographical systems fall into two categories**

| Geographical system | Sign | Measure | Mathematical methods | Geographical model (example) |
| --- | --- | --- | --- | --- |
| Scale systems | Characteristic length (typical scale) | Effective length, area, mean, standard deviation, eigenvalue, etc. | Calculus, linear algebra, probability theory and statistics, etc. | Clark model of population density |
| Scale-free systems | Scaling (no characteristic length) | No effective length, area, mean, standard deviation, eigenvalue, etc. | Fractal geometry, complex network theory, wavelet analysis, renormalization group, etc. | Smeed model of urban traffic network |

1. Gordon K (2005). The mysteries of mass. Scientific American. 293(1): 40-46/48.
2. Henry J (2002). The Scientific Revolution and the Origins of Modern Science (2nd Edition). New York: Palgrave
3. Feder J (1988). Fractals (physics of solids and liquids). New York: Plenu
4. Takayasu H (1990). Fractals in the Physical Sciences. Manchester: Manchester University Press
5. Hao BL (2004). Chaos and Fractals: a Collection of Popular Science Works by Hao Bolin. Shanghai: Shanghai Science and Technology Press (In Chinese)
6. Liu SD, Liu SK (1993). An Introduction to Fractals and Fractal Dimension. Beijing: China Meteorological Press (In Chinese)
7. Wang FQ, Li HQ (1996). *Fractals--The Artistic Structure of Nature*. Jinan: Shandong Education Press (In Chinese)

•**[Reviewer #2]** p. 2, L. 12-23: I do not agree with this view and I am not convinced by the statements of the author. First, the text is quite hard to follow. Please give examples of statistics/approaches with appropriate referecnces, and be clearer on the different methods described. For me, the spatio-temporal analogy is as follows: (i) correlations, temporal autocorrelation (von Neumann, 1941; von Neumann et al., 1941) and spatial autocorrelation (Geary, 1954; Moran, 1950) and (ii) autocorrelation functions (ACF, PACF) and correlograms, variograms. The first approaches looks at (dis)similarities between two values that can be separated by a lag (in time or space) while the second look at the same patterns but provides a functional description at different lag values. The spatial n*n weighting matrix (where n corresponds to the number of sampling units) corresponds to a matrix with the same dimensions that define that one observation is the neighbor (possibly weigted) of its precedent and subsequent observations (in a symmetric definition of neighboring, only subsequent for an asymmetric definition). As this part motivates the work, this comment should be carefully considered.

**[Reply]** Now, a paragraph and a table have been added to the revised version as follows:

As we know, the modeling methods of ACF and PACF as well as the related spectrums have been developed for time series analysis. A time series is in fact a 1-dimensional variable based on ordered point sets. The methods for time series analysis can be applied to 1-dimensional spatial series based on isotropic ordered spatial point sets. However, the methods cannot be directly generalized to the 2-dimensional spatial data based on anisotropic random spatial point sets (Table 3). Correlogram is a basic way for illustrating ACF and PACF in time series analysis. It is natural for this tool to be introduced into spatial autocorrelation analysis based on variable distance. However, the development of spatial autocorrelation function analysis is a system engineering. The methodology cannot be completely represented by correlograms and autocorrelation coefficients based on variable distance. Developing spatial autocorrelation functions relies heavily on three necessary conditions. First, introduction of spatial displacement parameter into spatial contiguity matrix. This step is easy to do, and, as mentioned above, many scholars have already done so (Bjørnstad and Falck, 2001; Getis and Ord, 1992; Legendre and Legendre, 1998; Ord and Getis, 1995). The key is to select a proper distance decay function. Second, definition of spatial contiguity matrix. This seems to be an easy problem to solve, but it is not. This involves the treatment of diagonal elements of the spatial weight matrix. Third, conversion of the spatial contiguity matrix to a weight matrix. If and only if the spatial contiguity matrix is normalized to yield a weight matrix, the key step can be revealed clearly, that is, the sum used to normalize the spatial contiguity matrix does not change over the spatial displacement parameter. Otherwise, the spatial autocorrelation functions cannot correspond to the temporal autocorrelation functions of time series analysis.

**Table 3 A comparison of the analytical processes of autocorrelation functions and related methods**

| Domain | Dimension | Object | ACF | PACF | Spectral analysis |
| --- | --- | --- | --- | --- | --- |
| Time domain | 1-dimension time | Time series | Temporal ACF analysis and auto-regressive process | Temporal PACF analysis and auto-regressive process | Power spectrum |
| Spatial domain | 1-dimension space | Isotropic ordered spatial series | Spatial ACF analysis and auto-regressive process | Spatial PACF analysis and auto-regressive process | Wave spectrum |
|  | 2-dimension space | Anisotropic random spatial series | To be developed | To be developed | To be developed |

The following context is used to illustrate the isotropy-based order 1-dimension spatial series:

**Table 1.** Four 1-dimensional ordered spatial series of urban population density based on isotropy assumption--Datasets of urban population density and distance by ring zones in Hangzhou in 1964, 1982, 1990, 2000, and 2010

| The order of rings  (*i*) | Distance  (km) | Population density (p/km^2^) | | | | |
| --- | --- | --- | --- | --- | --- | --- |
|  |  | 1964 | 1982 | 1990 | 2000 | 2010 |
| 1 | 0.3 | 24,131 | 29,540 | 29,928 | 28,184 | 26,635 |
| 2 | 0.9 | 18,966 | 22,225 | 26,634 | 26,821 | 25,419 |
| 3 | 1.5 | 16,282 | 18,957 | 22,262 | 24,621 | 22,702 |
| 4 | 2.1 | 16,007 | 19,232 | 21,612 | 23,176 | 20,923 |
| 5 | 2.7 | 13,052 | 15,439 | 17,290 | 18,910 | 19,466 |
| 6 | 3.3 | 8,259 | 9,929 | 12,478 | 16,911 | 18,830 |
| 7 | 3.9 | 5,798 | 7,026 | 9,896 | 14,522 | 16,594 |
| 8 | 4.5 | 2,626 | 3,461 | 5,560 | 10,829 | 12,428 |
| 9 | 5.1 | 2,143 | 2,807 | 4,180 | 7,282 | 9,226 |
| 10 | 5.7 | 2,142 | 2,689 | 3,923 | 6,200 | 7,996 |
| 11 | 6.3 | 2,185 | 2,566 | 3,516 | 5,644 | 7,363 |
| 12 | 6.9 | 1,438 | 1,693 | 2,197 | 4,297 | 6,487 |
| 13 | 7.5 | 1,083 | 1,371 | 1,796 | 3,806 | 5,863 |
| 14 | 8.1 | 967 | 1,256 | 1,634 | 3,153 | 5,260 |
| 15 | 8.7 | 842 | 1,114 | 1,442 | 2,683 | 4,830 |
| 16 | 9.3 | 848 | 973 | 1,265 | 2,354 | 4,509 |
| 17 | 9.9 | 818 | 1,051 | 1,163 | 2,028 | 4,200 |
| 18 | 10.5 | 812 | 1,051 | 1,143 | 1,828 | 4,062 |
| 19 | 11.1 | 807 | 1,051 | 1,160 | 1,651 | 3,846 |
| 20 | 11.7 | 625 | 979 | 1,093 | 1,581 | 3,788 |
| 21 | 12.3 | 691 | 901 | 1,006 | 1,490 | 3,673 |
| 22 | 12.9 | 575 | 870 | 972 | 1,465 | 3,543 |
| 23 | 13.5 | 532 | 666 | 817 | 1,278 | 2,953 |
| 24 | 14.1 | 381 | 487 | 679 | 1,033 | 2,630 |
| 25 | 14.7 | 369 | 489 | 582 | 958 | 2,351 |
| 26 | 15.3 | 375 | 456 | 563 | 882 | 2,125 |

**Figure 1.** A map of zonal system indicating sub-districts of Hangzhou and corresponding system of concentric circles. Note: For urbanized area, a sub-district is termed *jiedao* in Chinese, and for rural area, sub-district is termed *Xiang* and *Zhen*.

•**[Reviewer #2]** p. 4, L. last: The discussion about normalization is useless. I think that the author should focus on one subject and remove these parts.

**[Reply]** This has been removed now.

**[Reviewer #2]** Moreover, the author states that total normalization is often used but row-standardization (or normalization) is much more used in geography or ecology as it simplifies the interpretation (Moran's scatterplot Anselin, 1996)

**[Reply]** I am sure Anselin made a reasoning mistakes when he used row standardization. I will write a paper entitled “*Reconstruction and normalization of local spatial association index (LISA)*” to clarify this problem recently.

I firmly believe that standardization by row is a mistake in geographical spatial analysis. A spatial weight matrix is based on a distance matrix or a generalized distance matrix, which must follow distance axiom (nonnegativity, canonicity, symmetry, and three point inequality). The method of row-standardization violates the symmetry condition in the distance axiom. This results in the outlier values of Moran’s index.

Of course, this question is beyond the topic of this article.

•**[Reviewer #2]** p. 5: I do not understand what you want to explain here. Why not directly give the formula of eigenvalues/eigenvectors. Why do you need all this mathematical playing of matrix products?

**[Reply]** In the revised version, the uses of these equations have been illustrated. Equations (12) can be used to distinguish characteristic scale from scaling, and equations (14) and (16) can be employed to generate canonical Moran’s scatterplots.

Further explanations are as follows:

First, this equations are not mathematical playing. They are useful for more theoretical understanding than quantitative analysis. They involve important knowledge to distinguish scaling analysis from the analysis method based on characteristic scale.

Second, this paper is not an isolated article. Instead, it is one of the companion papers. Another related paper is entitled “Derivation of correlation dimension from spatial autocorrelation functions”. These equations will be helpful for the readers who like both spatial autocorrelation analysis and fractal modeling. My research field is basic theory rather than quantitative analysis, but I have been teaching the methods of quantitative analyses for geography.

- Chen YG (2019). Derivation of correlation dimension from spatial autocorrelation functions. *arXiv:1901.03837* [physics.soc-ph], https://arxiv.org/abs/1901.03837

To response to reviewer #2 and reviewer#3, a paragraph has been added to Section 3 as below:

The autocorrelation functions based on Moran’s index involves negative values, and cannot be directly associated with scaling relation. The solution to a scaling equation is always a power law. So power law is the basic mark of scaling in positive studies. In equation (12), if **T** represents a contraction-dilation transformation, and a function satisfies equation (12), we will say it follows the scaling law. The values of Geary’s coefficient and Getis-Ord’s index are greater than 0 in empirical studies and may follow a power law. For the autocorrelation function based on Geary’s coefficient, the power law relation is as below

$C(r)=\frac{n-1}{n}[e^{\text{T}}W(r)z^{2}-I(r)]=C_{0}r^{a}$, (43)

where *C*_0_ refers to the proportionality coefficient, and *a* to a scaling exponent. For the Getis-Ord’s index, the power law relation is as follows

$G(r)=y^{\text{T}}W(r)y=G_{0}r^{b}$, (44)

in which *G*_0_ refers to the proportionality coefficient, and *b* to a scaling exponent.

Besides Moran’s index, the changing feature of spatial autocorrelation can be reflected by Moran’s scatterplots. Based on spatial autocorrelation functions, a series of canonical Moran’s scatterplots can be drawn by means of equation (14) and (16). These graphs can reflect the positive and negative alternation process and local characteristics of spatial autocorrelation (Figure 7).

nWz, zzTWz

| z | nWz | zz'Wz |
| --- | --- | --- |
| 2.75231 | -0.0310278 | -0.0117561 |
| 1.02329 | 0 | -0.00437085 |
| -0.551633 | 0 | 0.00235623 |
| -0.473587 | 0.0841827 | 0.00202286 |
| -0.892537 | 0 | 0.00381236 |
| 0.199551 | -0.00214316 | -0.000852358 |
| -0.413978 | 0 | 0.00176825 |
| -0.0600085 | 0.00712683 | 0.000256319 |
| 3.29157 | 0 | -0.0140595 |
| 0.192078 | 0 | -0.000820438 |
| -0.124427 | -0.0167557 | 0.000531473 |
| -0.46916 | -0.0318861 | 0.00200395 |
| -0.572727 | 0 | 0.00244633 |
| -0.768384 | -0.0167557 | 0.00328205 |
| -0.39519 | 0.0813829 | 0.001688 |
| -0.326739 | 0.0219227 | 0.00139563 |
| 0.616192 | -0.0116693 | -0.00263198 |
| -0.520628 | 0 | 0.00222379 |
| 1.12319 | 0 | -0.00479757 |
| -0.637482 | 0 | 0.00272292 |
| 0.975464 | -0.0134896 | -0.00416657 |
| 0.258203 | 0.034838 | -0.00110288 |
| -0.635912 | 0.034838 | 0.00271622 |
| -0.451795 | 0 | 0.00192979 |
| -0.00235544 | -0.0116693 | 1.00609e-05 |
| -0.648645 | -0.0347933 | 0.0027706 |
| -0.968848 | 0 | 0.00413831 |
| -0.974212 | -0.0231659 | 0.00416122 |
| -0.543597 | 0 | 0.00232191 |

nWz, zzTWz

| z | nWz | zz'Wz |
| --- | --- | --- |
| 2.75231 | -0.0167557 | -0.0010227 |
| 1.02329 | 0 | -0.000380232 |
| -0.551633 | 0.00712683 | 0.000204975 |
| -0.473587 | -0.0167557 | 0.000175975 |
| -0.892537 | -0.0231659 | 0.000331648 |
| 0.199551 | -0.0338151 | -7.4149e-05 |
| -0.413978 | 0 | 0.000153825 |
| -0.0600085 | 0 | 2.22979e-05 |
| 3.29157 | 0 | -0.00122308 |
| 0.192078 | 0 | -7.13722e-05 |
| -0.124427 | -0.0257832 | 4.62343e-05 |
| -0.46916 | 0.0813829 | 0.00017433 |
| -0.572727 | 0 | 0.000212813 |
| -0.768384 | -0.0141139 | 0.000285515 |
| -0.39519 | -0.0247593 | 0.000146844 |
| -0.326739 | -0.00444381 | 0.000121409 |
| 0.616192 | 0.040114 | -0.000228964 |
| -0.520628 | 0 | 0.000193454 |
| 1.12319 | 0.0220069 | -0.000417354 |
| -0.637482 | 0 | 0.000236874 |
| 0.975464 | -0.0161355 | -0.000362461 |
| 0.258203 | -0.0161355 | -9.59427e-05 |
| -0.635912 | 0 | 0.000236291 |
| -0.451795 | 0.0440595 | 0.000167877 |
| -0.00235544 | 0 | 8.75229e-07 |
| -0.648645 | -0.0318763 | 0.000241022 |
| -0.968848 | 0 | 0.000360003 |
| -0.974212 | 0 | 0.000361996 |
| -0.543597 | 0 | 0.000201989 |

(a) 450-550 km	(b) 1050-1150 km

nWz, zzTWz

| z | nWz | zz'Wz |
| --- | --- | --- |
| 2.75231 | 0.117556 | 0.0547466 |
| 1.02329 | -0.0392371 | 0.0203544 |
| -0.551633 | -0.0347933 | -0.0109726 |
| -0.473587 | 0.0461398 | -0.0094202 |
| -0.892537 | 0 | -0.0177536 |
| 0.199551 | -0.0116693 | 0.00396931 |
| -0.413978 | 0 | -0.0082345 |
| -0.0600085 | 0 | -0.00119364 |
| 3.29157 | 0.0812987 | 0.0654732 |
| 0.192078 | 0 | 0.00382067 |
| -0.124427 | 0.036546 | -0.00247499 |
| -0.46916 | 0 | -0.00933214 |
| -0.572727 | 0 | -0.0113922 |
| -0.768384 | -0.0169138 | -0.0152841 |
| -0.39519 | 0 | -0.0078608 |
| -0.326739 | 0.00712683 | -0.00649923 |
| 0.616192 | 0 | 0.0122568 |
| -0.520628 | -0.0169138 | -0.0103559 |
| 1.12319 | 0 | 0.0223416 |
| -0.637482 | -0.0161355 | -0.0126803 |
| 0.975464 | 0 | 0.0194031 |
| 0.258203 | -0.0169138 | 0.00513597 |
| -0.635912 | 0 | -0.012649 |
| -0.451795 | -0.0227672 | -0.00898674 |
| -0.00235544 | 0.117556 | -4.68524e-05 |
| -0.648645 | 0 | -0.0129023 |
| -0.968848 | -0.0169138 | -0.0192715 |
| -0.974212 | 0.0168448 | -0.0193782 |
| -0.543597 | 0 | -0.0108128 |

nWz, zzTWz

| z | nWz | zz'Wz |
| --- | --- | --- |
| 2.75231 | -0.0227672 | -0.0169141 |
| 1.02329 | 0 | -0.00628853 |
| -0.551633 | 0 | 0.00339001 |
| -0.473587 | -0.0161355 | 0.00291038 |
| -0.892537 | 0.034838 | 0.00548501 |
| 0.199551 | -0.0231659 | -0.00122632 |
| -0.413978 | -0.0185939 | 0.00254406 |
| -0.0600085 | 0.0827628 | 0.000368777 |
| 3.29157 | -0.00214316 | -0.0202281 |
| 0.192078 | 0 | -0.0011804 |
| -0.124427 | 0.00922154 | 0.000764653 |
| -0.46916 | 0 | 0.00288318 |
| -0.572727 | 0 | 0.00351964 |
| -0.768384 | 0 | 0.00472204 |
| -0.39519 | 0 | 0.00242861 |
| -0.326739 | 0 | 0.00200795 |
| 0.616192 | 0 | -0.00378675 |
| -0.520628 | -0.0147849 | 0.00319947 |
| 1.12319 | 0 | -0.00690247 |
| -0.637482 | 0.0982967 | 0.00391759 |
| 0.975464 | -0.0318763 | -0.00599462 |
| 0.258203 | -0.00444381 | -0.00158676 |
| -0.635912 | 0 | 0.00390794 |
| -0.451795 | -0.0169138 | 0.00277647 |
| -0.00235544 | -0.0194142 | 1.44751e-05 |
| -0.648645 | 0.00712683 | 0.00398619 |
| -0.968848 | 0 | 0.00595397 |
| -0.974212 | -0.00214316 | 0.00598693 |
| -0.543597 | -8.41227e-05 | 0.00334063 |

(c) 1450-1550 km	(d) 2550-2650 km

**Figure 7 The canonical Moran’s scatterplots of spatial autocorrelation based on correlation density function for the main cities of China (examples for 2010)**

(**Note**: The scatter points are based on the inner product correlation, *z*^T^*zWz*=*Iz*, and the relation is equation (14). The trend line is based on the outer product correlation, *zz***^T^***Wz*=*Iz*, and the relation is equation (16). The Moran’s index difference values are as follows. (a) For 450<*r*≤550, ∆*I*=-0.0043; (b) For 1050<*r*≤1150, ∆*I*=-0.0004; (c) For 1450<*r*≤1550, ∆*I*=0.0199; (d) For 2550<*r*≤2650, ∆*I*=0.0061.)

•**[Reviewer #2]** there are a lot of statements in the paper that are more opininions than reality (e.g., "A good model is ..." (P. 6, L. 2). Please avoid this type of sentences.

**[Reply]** I quite agree with you that there are should not be more opinions than reality in a paper. I revised these types of expressions, but I reserve my opinion.

In fact, the expression such as “a good model” came from well-known Chinese theoretical physical scientist, Academician Bolin Hao (1986), and so on. This involves a significant fact, scientific research transition. After World War II, scientific research gradually transformed from truth finding to model building. As John von Neumann (1961) once said: “*The sciences do not try to explain, they hardly even try to interpret, they mainly make models.*”

Models are different from truth. Generally speaking, models fall into the good and the bad rather than the right and the wrong. As for positive studies, as Gabaix and Ioannides said, “*The main question of empirical work should be how well a theory fits, rather than whether or not it fits perfectly (i.e., within the standard errors).*”

- von Neumann J (1961). *Collected works (Vol.6)*. New York/Oxford: Pergamon Press, page 492
- Hao BL (1986). Fractals and fractal dimensions. *Science*, 38 (1): 9-17 (in Chinese)
- Gabaix X, Ioannides YM (2004). The evolution of city size distributions. In: *Handbook of Urban and Regional Economics, Volume 4* (Chapter 53). Eds. J. V. Henderson and J. F. Thisse. Amsterdam: North-Holland Publishing Company, pp 2341-2378.

•**[Reviewer #2]** bibliography is really centred on some publications (except in the discussion) but a lot of very traditional/approaches works are ignored and not cited (correlograms, row standardization of the spatial weighting matrix) during the methodological description of methods.

**[Reply]** I have cited the papers and books recommended by you. Thank you very much.

PS: Adding line numbering would be appreciated to facilitate the reviewing process.

**[Reply]** I will add line numbering in the revised version.

Cheers

#### References

Anselin, L. (1996). The Moran scatterplot as an ESDA tool to assess local instability in spatial association. In Fischer, M. M., Scholten, H. J., and Unwin, D., editors, *Spatial analytical perspectives on GIS*, pages 111-125.Taylor and Francis, London.

de Jong, P., Sprenger, C., and van Veen, F. (1984). On extreme values of Moran's I and Geary's C. *Geographical Analysis*, 16(1):17-24.

Dray, S. (2011). A new perspective about Moran's coefficient: spatial auto-correlation as a linear regression problem. *Geographical Analysis*, 43:127-141.

Dray, S., Legendre, P., and Peres-Neto, P. R. (2006). Spatial modeling: a comprehensive framework for principal coordinate analysis of neighbor matrices (PCNM). *Ecological Modelling*, 196:483-493.

Geary, R. C. (1954). The contiguity ratio and statistical mapping. *The Incorporated Statistician*, 5(3):115-145.

Griffith, D. A. (1996). Spatial autocorrelation and eigenfunctions of the geographic weights matrix accompanying geo-referenced data. *Canadian Geographer*, 40(4): 351-367.

Griffith, D. A. (2003). *Spatial Autocorrelation and Spatial Filtering: Gaining Understanding through Theory and Scientific Visualization*. Springer-Verlag, Berlin.

Legendre, P. and Legendre, L. (1998). *Numerical Ecology*. Elsevier Science, Amsterdam, second edition.

Moran, P. A. P. (1950). Notes on continuous stochastic phenomena. *Biometrika*, 37:17-23.

von Neumann, J. (1941). Distribution of the ratio of the mean square successive difference to the variance. *The Annals of Mathematical Statistics*, 12:367-395.

von Neumann, J., Kent, R. H., Bellinson, H. R., and Hart, B. I. (1941). The mean square successive difference. *The Annals of Mathematical Statistics*, 12:153-162.

### Reviewer #3

**[Reviewer #3]** Spatial autocorrelation is one of the most important methods in quantitative analyses of geography. This research proposed a new analytical framework based on a series of spatial autocorrelation functions, which, I believe, has made significant contributions both theoretically and practically. However, before publication, I think some points about the empirical analysis need to be clarified or discussed.

**[Reviewer #3]**1. Only 29 capital cities were included in the analysis. Why not perform the analysis at a city level? I think it might be more appropriate for reflecting “scale”.

**[Reply]** I quite agree with you, the spatial sample should be determined by “scale”. The reasons for using 29 provincial capital cities in China as research samples are as follows:

First, research objectives. The aim of this paper at developing theory and methodology for spatial autocorrelation analysis. This case was mainly employed to demonstrate the application of method rather than empirical analysis. In this instance, the example cannot be too complex.

Second, data limitation. The spatial distance data based on railway come from traffic atlas which has been published. This data was adopted so that readers can easily find the same data and test the method proposed in this paper.

Third, relative studies. In the previously published papers, the case studies were based on the 29 provincial capital cities of China. For the sake of reference and comparability for the cases based on different methods, this paper is still based on the 29 cities.

Now, a paragraph has been added to the Discussion to clarify this shortcoming as below:

“**Third, the case study is based on 29 provincial capital cities rather than a system of cities based on certain size threshold.** The system of provincial capital cities are in the administrative sense instead of pure geographical sense. This type of spatial sample can be used to generate example to illustrate a research method. If we perform a spatial analysis of Chinese cities, we should make a spatial sampling according to certain scale threshold. Due to limitation of space, the problems remain to be further solved in future studies.”

**[Reviewer #3]**2. I don’t think Haikou should be excluded because it was linked to other capital cities like Guangzhou by train even there is no real railway between Guangdong Province and Haikou.

**[Reply]** I agree with you. I hope to take Haikou into account in this case study. However, as indicated above, the distance matrix came from a traffic atlas, in which Haikou was excluded. In other words, even if we find the distance from Haikou to Guangzhou, but we cannot find the distances from Haikou to other cities in China. The lengths of roads and railways depend on measurement scales. The lengths of railways provided by different organizations or departments are not necessarily comparable.

In fact, I plan to have one of my students make an empirical research on Chinese cities by means of spatial autocorrelation analysis and interaction modeling. The spatial sample will be determined by city size threshold, and spatial distance matrix will be obtained by ArcGIS technology.

**[Reviewer #3]**3. Only functions based on Moran’s Index were reported in subsection 3.2. How about other indexes? Will the extent to which the value of *r* influence the functions vary by index?

**[Reply]** The spatial autocorrelation functions based on Geary’s coefficient and Getis-Ord’s index involve scaling analysis. I originally planning to discuss it another paper. In the revised version, the spatial autocorrelation functions based on Geary’s *C* and Getis’s *G* has been taken into account. A subsection titled “Scaling analysis of spatial autocorrelation” has been added to the Section 3.

### 3.3 Scaling analysis of spatial autocorrelation

The autocorrelation functions based on Moran’s index involves negative values, and cannot be directly associated with scaling relation. The solution to a scaling equation is always a power law. So power law is the basic mark of scaling in positive studies. In equation (12), if **T** represents a contraction-dilation transformation, and a function satisfies equation (12), we will say it follows the scaling law. The values of Geary’s coefficient and Getis-Ord’s index are greater than 0 in empirical studies and may follow a power law. For the autocorrelation function based on Geary’s coefficient, the power law relation is as below

$C(r)=\frac{n-1}{n}[e^{\text{T}}W(r)z^{2}-I(r)]=C_{0}r^{a}$, (43)

where *C*_0_ refers to the proportionality coefficient, and *a* to a scaling exponent. For the Getis-Ord’s index, the power law relation is as follows

$G(r)=y^{\text{T}}W(r)y=G_{0}r^{b}$, (44)

in which *G*_0_ refers to the proportionality coefficient, and *b* to a scaling exponent. The empirical analyses show that both the spatial cumulative autocorrelation function based on Geary’s coefficient and that based on Getis-Ord’s index follow power law if scaling range is taken into account (Figure 8, Figure 9). For Geary’s coefficient, the scaling ranges from 250 to 2750 km. The scaling exponent changed from 1.5479 in 2000 to 1.5863 in 2010 (Figure 8). For Getis-Ord’s index, the scaling range come between 150 and 2750 km. The scaling exponent turned from 1.5811 in 2000 to 1.4986 in 2010 (Figure 9).

Geary's C (C(r)

| Yardstick (r) | Geary C | Scaling range |
| --- | --- | --- |
| 150 | 0.00293941 |  |
| 250 | 0.0209697 | 0.0209697 |
| 350 | 0.0462237 | 0.0462237 |
| 450 | 0.0569734 | 0.0569734 |
| 550 | 0.0797608 | 0.0797608 |
| 650 | 0.103328 | 0.103328 |
| 750 | 0.133216 | 0.133216 |
| 850 | 0.160326 | 0.160326 |
| 950 | 0.166087 | 0.166087 |
| 1050 | 0.223632 | 0.223632 |
| 1150 | 0.238464 | 0.238464 |
| 1250 | 0.313847 | 0.313847 |
| 1350 | 0.384683 | 0.384683 |
| 1450 | 0.407594 | 0.407594 |
| 1550 | 0.447743 | 0.447743 |
| 1650 | 0.480468 | 0.480468 |
| 1750 | 0.494694 | 0.494694 |
| 1850 | 0.528501 | 0.528501 |
| 1950 | 0.553154 | 0.553154 |
| 2050 | 0.574064 | 0.574064 |
| 2150 | 0.678116 | 0.678116 |
| 2250 | 0.716569 | 0.716569 |
| 2350 | 0.736615 | 0.736615 |
| 2450 | 0.81875 | 0.81875 |
| 2550 | 0.842304 | 0.842304 |
| 2650 | 0.875005 | 0.875005 |
| 2750 | 0.89824 | 0.89824 |
| 2850 | 0.90549 |  |
| 2950 | 0.906907 |  |
| 3050 | 0.927333 |  |
| 3150 | 0.93049 |  |
| 3250 | 0.939787 |  |
| 3350 | 0.947069 |  |
| 3450 | 0.949034 |  |
| 3550 | 0.952025 |  |
| 3650 | 0.958334 |  |
| 3750 | 0.958469 |  |
| 3850 | 0.969549 |  |
| 3950 | 0.973036 |  |
| 4050 | 0.973064 |  |
| 4150 | 0.993547 |  |
| 4250 | 0.993555 |  |
| 4350 | 0.993555 |  |
| 4450 | 0.993556 |  |
| 4550 | 0.994867 |  |
| 4650 | 0.994867 |  |
| 4750 | 0.999307 |  |
| 4850 | 0.999465 |  |
| 4950 | 0.999465 |  |
| 5050 | 0.999499 |  |
| 5150 | 1 |  |

Geary's C (C(r)

| Yardstick (r) | Geary C | Scaling range |
| --- | --- | --- |
| 150 | 0.0035547 |  |
| 250 | 0.017708 | 0.017708 |
| 350 | 0.0431076 | 0.0431076 |
| 450 | 0.0525178 | 0.0525178 |
| 550 | 0.0820141 | 0.0820141 |
| 650 | 0.102339 | 0.102339 |
| 750 | 0.142681 | 0.142681 |
| 850 | 0.171113 | 0.171113 |
| 950 | 0.174627 | 0.174627 |
| 1050 | 0.232663 | 0.232663 |
| 1150 | 0.249794 | 0.249794 |
| 1250 | 0.324357 | 0.324357 |
| 1350 | 0.39197 | 0.39197 |
| 1450 | 0.421649 | 0.421649 |
| 1550 | 0.459681 | 0.459681 |
| 1650 | 0.494116 | 0.494116 |
| 1750 | 0.506849 | 0.506849 |
| 1850 | 0.542367 | 0.542367 |
| 1950 | 0.561691 | 0.561691 |
| 2050 | 0.58178 | 0.58178 |
| 2150 | 0.684615 | 0.684615 |
| 2250 | 0.723171 | 0.723171 |
| 2350 | 0.747689 | 0.747689 |
| 2450 | 0.819955 | 0.819955 |
| 2550 | 0.844002 | 0.844002 |
| 2650 | 0.877557 | 0.877557 |
| 2750 | 0.901885 | 0.901885 |
| 2850 | 0.908782 |  |
| 2950 | 0.909821 |  |
| 3050 | 0.927011 |  |
| 3150 | 0.930767 |  |
| 3250 | 0.943645 |  |
| 3350 | 0.951264 |  |
| 3450 | 0.953521 |  |
| 3550 | 0.956577 |  |
| 3650 | 0.959959 |  |
| 3750 | 0.959992 |  |
| 3850 | 0.973947 |  |
| 3950 | 0.977767 |  |
| 4050 | 0.977778 |  |
| 4150 | 0.995486 |  |
| 4250 | 0.995488 |  |
| 4350 | 0.995488 |  |
| 4450 | 0.995548 |  |
| 4550 | 0.996387 |  |
| 4650 | 0.996387 |  |
| 4750 | 0.99969 |  |
| 4850 | 0.99971 |  |
| 4950 | 0.99971 |  |
| 5050 | 0.999722 |  |
| 5150 | 1 |  |

a. 2000	b. 2010

**Figure 8 The scaling relations for the spatial autocorrelation function based on cumulative correlation and Geary’s coefficient**

**Note:** The solid dots represent all points of spatial autocorrelation functions, and the hollow blocks represent the points within the scaling range. The scaling range comes between 250 and 2750 km.

Getis-ord's G (G(r)

| Yardstick (r) | Geary C | Scaling range |
| --- | --- | --- |
| 150 | 1.27049e-05 | 1.27049e-05 |
| 250 | 2.45255e-05 | 2.45255e-05 |
| 350 | 4.56578e-05 | 4.56578e-05 |
| 450 | 6.2194e-05 | 6.2194e-05 |
| 550 | 9.6136e-05 | 9.6136e-05 |
| 650 | 0.000111317 | 0.000111317 |
| 750 | 0.000149806 | 0.000149806 |
| 850 | 0.000165299 | 0.000165299 |
| 950 | 0.000175238 | 0.000175238 |
| 1050 | 0.000229029 | 0.000229029 |
| 1150 | 0.000260346 | 0.000260346 |
| 1250 | 0.000374818 | 0.000374818 |
| 1350 | 0.000440863 | 0.000440863 |
| 1450 | 0.000476232 | 0.000476232 |
| 1550 | 0.000539767 | 0.000539767 |
| 1650 | 0.000578461 | 0.000578461 |
| 1750 | 0.000599543 | 0.000599543 |
| 1850 | 0.000656503 | 0.000656503 |
| 1950 | 0.000681503 | 0.000681503 |
| 2050 | 0.000730628 | 0.000730628 |
| 2150 | 0.000802728 | 0.000802728 |
| 2250 | 0.00084855 | 0.00084855 |
| 2350 | 0.000892395 | 0.000892395 |
| 2450 | 0.000932138 | 0.000932138 |
| 2550 | 0.000983205 | 0.000983205 |
| 2650 | 0.00100838 | 0.00100838 |
| 2750 | 0.00102673 | 0.00102673 |
| 2850 | 0.0010431 |  |
| 2950 | 0.00104956 |  |
| 3050 | 0.00106335 |  |
| 3150 | 0.00107755 |  |
| 3250 | 0.00108737 |  |
| 3350 | 0.00110337 |  |
| 3450 | 0.00110905 |  |
| 3550 | 0.00111408 |  |
| 3650 | 0.00112507 |  |
| 3750 | 0.00112683 |  |
| 3850 | 0.00113405 |  |
| 3950 | 0.00114025 |  |
| 4050 | 0.00114197 |  |
| 4150 | 0.00114965 |  |
| 4250 | 0.00115133 |  |
| 4350 | 0.00115133 |  |
| 4450 | 0.00115206 |  |
| 4550 | 0.00115611 |  |
| 4650 | 0.00115611 |  |
| 4750 | 0.00115909 |  |
| 4850 | 0.00116027 |  |
| 4950 | 0.00116027 |  |
| 5050 | 0.00116172 |  |
| 5150 | 0.00116322 |  |

Getis-ord's G (G(r)

| Yardstick (r) | Geary C | Scaling range |
| --- | --- | --- |
| 150 | 1.68473e-05 | 1.68473e-05 |
| 250 | 2.96055e-05 | 2.96055e-05 |
| 350 | 5.25488e-05 | 5.25488e-05 |
| 450 | 6.83867e-05 | 6.83867e-05 |
| 550 | 0.000102025 | 0.000102025 |
| 650 | 0.000119099 | 0.000119099 |
| 750 | 0.000158818 | 0.000158818 |
| 850 | 0.000173953 | 0.000173953 |
| 950 | 0.000183107 | 0.000183107 |
| 1050 | 0.00023565 | 0.00023565 |
| 1150 | 0.000265703 | 0.000265703 |
| 1250 | 0.000370985 | 0.000370985 |
| 1350 | 0.000440047 | 0.000440047 |
| 1450 | 0.000473646 | 0.000473646 |
| 1550 | 0.000538586 | 0.000538586 |
| 1650 | 0.000579026 | 0.000579026 |
| 1750 | 0.000598351 | 0.000598351 |
| 1850 | 0.000655334 | 0.000655334 |
| 1950 | 0.000679314 | 0.000679314 |
| 2050 | 0.000729791 | 0.000729791 |
| 2150 | 0.000804259 | 0.000804259 |
| 2250 | 0.000850522 | 0.000850522 |
| 2350 | 0.000895625 | 0.000895625 |
| 2450 | 0.000935597 | 0.000935597 |
| 2550 | 0.000984671 | 0.000984671 |
| 2650 | 0.00101033 | 0.00101033 |
| 2750 | 0.00102849 | 0.00102849 |
| 2850 | 0.00104399 |  |
| 2950 | 0.0010495 |  |
| 3050 | 0.00106259 |  |
| 3150 | 0.00107589 |  |
| 3250 | 0.00108567 |  |
| 3350 | 0.0011007 |  |
| 3450 | 0.0011063 |  |
| 3550 | 0.00111172 |  |
| 3650 | 0.00112142 |  |
| 3750 | 0.00112362 |  |
| 3850 | 0.00113223 |  |
| 3950 | 0.00113881 |  |
| 4050 | 0.00114062 |  |
| 4150 | 0.00114921 |  |
| 4250 | 0.00115048 |  |
| 4350 | 0.00115048 |  |
| 4450 | 0.00115113 |  |
| 4550 | 0.00115486 |  |
| 4650 | 0.00115486 |  |
| 4750 | 0.00115801 |  |
| 4850 | 0.00115913 |  |
| 4950 | 0.00115913 |  |
| 5050 | 0.00116087 |  |
| 5150 | 0.00116246 |  |

a. 2000	b. 2010

**Figure 9 The scaling relations for the spatial autocorrelation function based on cumulative correlation and Getis-Ord’s index**

**Note:** The solid dots represent all points of spatial autocorrelation functions, and the hollow blocks represent the points within the scaling range. The scaling range comes between 150 and 2750 km.

Using difference function, we can transform the cumulative autocorrelation functions based on Geary’s coefficient and Getis-Ord’s index into density autocorrelation functions. For Geary’s coefficient, the formula for the autocorrelation function based on density correlation is as follows

$C_{d}(r)=\left\{\begin{matrix} C(r_{k}),\text{ }k=1 \\ \Delta C(r_{k})=C(r_{k})-C(r_{k-1}),\text{ }k>1 \end{matrix}\right.$, (45)

For Getis-Ord’s coefficient, the formula for the autocorrelation function based on density correlation is as below

$G_{d}(r)=\left\{\begin{matrix} G(r_{k})=y^{\text{T}}W(r_{k})y,\text{ }k=1 \\ \Delta G(r_{k})=G(r_{k})-G(r_{k-1}),\text{ }k>1 \end{matrix}\right.$, (46)

In equations (45) and (46), the distance *r* is discretized as *r_k_*=*r*_0_+*ks*, in which *k*=1, 2, 3,…,*m* represents natural numbers, *s* refers to step length, and *r*_0_ is a constant.

Geary's C

| Distance (km) | dC2000 |
| --- | --- |
| 150 | 0.00293941 |
| 250 | 0.0180303 |
| 350 | 0.025254 |
| 450 | 0.0107497 |
| 550 | 0.0227873 |
| 650 | 0.023567 |
| 750 | 0.0298887 |
| 850 | 0.0271099 |
| 950 | 0.00576085 |
| 1050 | 0.0575449 |
| 1150 | 0.0148314 |
| 1250 | 0.0753838 |
| 1350 | 0.0708352 |
| 1450 | 0.0229117 |
| 1550 | 0.0401488 |
| 1650 | 0.0327248 |
| 1750 | 0.0142264 |
| 1850 | 0.0338071 |
| 1950 | 0.0246524 |
| 2050 | 0.02091 |
| 2150 | 0.104053 |
| 2250 | 0.038453 |
| 2350 | 0.0200453 |
| 2450 | 0.0821353 |
| 2550 | 0.0235542 |
| 2650 | 0.0327014 |
| 2750 | 0.0232347 |
| 2850 | 0.00724954 |
| 2950 | 0.00141771 |
| 3050 | 0.0204253 |
| 3150 | 0.00315762 |
| 3250 | 0.00929633 |
| 3350 | 0.00728233 |
| 3450 | 0.00196508 |
| 3550 | 0.00299125 |
| 3650 | 0.00630819 |
| 3750 | 0.000135535 |
| 3850 | 0.01108 |
| 3950 | 0.00348661 |
| 4050 | 2.7975e-05 |
| 4150 | 0.0204838 |
| 4250 | 7.97265e-06 |
| 4350 | 0 |
| 4450 | 5.65767e-07 |
| 4550 | 0.0013109 |
| 4650 | 0 |
| 4750 | 0.00444004 |
| 4850 | 0.000157646 |
| 4950 | 0 |
| 5050 | 3.41741e-05 |
| 5150 | 0.000501245 |

Geary's C

| Distance (km) | dC2010 |
| --- | --- |
| 150 | 0.0035547 |
| 250 | 0.0141533 |
| 350 | 0.0253995 |
| 450 | 0.00941024 |
| 550 | 0.0294963 |
| 650 | 0.0203251 |
| 750 | 0.0403423 |
| 850 | 0.0284317 |
| 950 | 0.00351402 |
| 1050 | 0.0580358 |
| 1150 | 0.0171309 |
| 1250 | 0.0745635 |
| 1350 | 0.0676127 |
| 1450 | 0.0296793 |
| 1550 | 0.0380314 |
| 1650 | 0.0344355 |
| 1750 | 0.0127328 |
| 1850 | 0.035518 |
| 1950 | 0.0193239 |
| 2050 | 0.0200892 |
| 2150 | 0.102835 |
| 2250 | 0.0385559 |
| 2350 | 0.0245184 |
| 2450 | 0.0722658 |
| 2550 | 0.024047 |
| 2650 | 0.0335548 |
| 2750 | 0.024328 |
| 2850 | 0.00689713 |
| 2950 | 0.00103879 |
| 3050 | 0.0171898 |
| 3150 | 0.00375627 |
| 3250 | 0.0128778 |
| 3350 | 0.00761907 |
| 3450 | 0.00225707 |
| 3550 | 0.00305647 |
| 3650 | 0.00338203 |
| 3750 | 3.27772e-05 |
| 3850 | 0.0139547 |
| 3950 | 0.00382029 |
| 4050 | 1.07606e-05 |
| 4150 | 0.0177083 |
| 4250 | 1.70058e-06 |
| 4350 | 0 |
| 4450 | 6.00822e-05 |
| 4550 | 0.0008392 |
| 4650 | 0 |
| 4750 | 0.00330343 |
| 4850 | 1.99778e-05 |
| 4950 | 0 |
| 5050 | 1.14897e-05 |
| 5150 | 0.000278072 |

a. 2000	b. 2010

**Figure 10 The curves of Geary’s *C* based on correlation density for the main cities of China**

(**Note**: Inside the scaling ranges of spatial correlation dimension, the curves of Geary’s *C* fluctuate sharply. From 2000 to 2010, the curve shapes have no significant change.)

The autocorrelation function based on density correlation can reflect the scaling range of spatial dependence from another view of angle. The density correlation function curves are a random fluctuation curves, and the autocorrelation function curve based on Geary’s coefficient looks like that based on Getis-Ord’s index (Figure 10, Figure 11). Within the scaling range, spatial correlation changes greatly with distance, while outside the scaling range, spatial correlation changes slightly over distance. This feature is similar to the change trend of the density autocorrelation function based on Moran’s index. The scaling range suggested by Geary’s coefficient and Getis-Ord’s index corresponds to that suggested by Moran’s index (150 or 250 km -2750 or 3350 km). It is hard to clarify the whole questions about spatial scaling in the autocorrelation processes by means of several paragraph of words, and the related problems will be discussed in a companion paper.

Getis-Ord's G

| Distance (km) | dG2000 |
| --- | --- |
| 150 | 1.27049e-05 |
| 250 | 1.18206e-05 |
| 350 | 2.11323e-05 |
| 450 | 1.65361e-05 |
| 550 | 3.3942e-05 |
| 650 | 1.51806e-05 |
| 750 | 3.84896e-05 |
| 850 | 1.54926e-05 |
| 950 | 9.93931e-06 |
| 1050 | 5.3791e-05 |
| 1150 | 3.13167e-05 |
| 1250 | 0.000114472 |
| 1350 | 6.60447e-05 |
| 1450 | 3.5369e-05 |
| 1550 | 6.35353e-05 |
| 1650 | 3.86938e-05 |
| 1750 | 2.10819e-05 |
| 1850 | 5.69603e-05 |
| 1950 | 2.49996e-05 |
| 2050 | 4.91255e-05 |
| 2150 | 7.20995e-05 |
| 2250 | 4.58224e-05 |
| 2350 | 4.38446e-05 |
| 2450 | 3.97433e-05 |
| 2550 | 5.10671e-05 |
| 2650 | 2.51786e-05 |
| 2750 | 1.83503e-05 |
| 2850 | 1.637e-05 |
| 2950 | 6.45089e-06 |
| 3050 | 1.37987e-05 |
| 3150 | 1.41981e-05 |
| 3250 | 9.81729e-06 |
| 3350 | 1.59982e-05 |
| 3450 | 5.68104e-06 |
| 3550 | 5.0322e-06 |
| 3650 | 1.09898e-05 |
| 3750 | 1.76116e-06 |
| 3850 | 7.22186e-06 |
| 3950 | 6.20104e-06 |
| 4050 | 1.71924e-06 |
| 4150 | 7.67771e-06 |
| 4250 | 1.68265e-06 |
| 4350 | 0 |
| 4450 | 7.26282e-07 |
| 4550 | 4.04603e-06 |
| 4650 | 0 |
| 4750 | 2.98778e-06 |
| 4850 | 1.1729e-06 |
| 4950 | 0 |
| 5050 | 1.45095e-06 |
| 5150 | 1.50289e-06 |

Getis-Ord's G

| Distance (km) | dG2010 |
| --- | --- |
| 150 | 1.68473e-05 |
| 250 | 1.27582e-05 |
| 350 | 2.29432e-05 |
| 450 | 1.5838e-05 |
| 550 | 3.36384e-05 |
| 650 | 1.70737e-05 |
| 750 | 3.97189e-05 |
| 850 | 1.51356e-05 |
| 950 | 9.15346e-06 |
| 1050 | 5.25428e-05 |
| 1150 | 3.00537e-05 |
| 1250 | 0.000105281 |
| 1350 | 6.90625e-05 |
| 1450 | 3.35986e-05 |
| 1550 | 6.49407e-05 |
| 1650 | 4.04398e-05 |
| 1750 | 1.93251e-05 |
| 1850 | 5.69821e-05 |
| 1950 | 2.39805e-05 |
| 2050 | 5.04773e-05 |
| 2150 | 7.44673e-05 |
| 2250 | 4.62633e-05 |
| 2350 | 4.51029e-05 |
| 2450 | 3.99721e-05 |
| 2550 | 4.90737e-05 |
| 2650 | 2.5661e-05 |
| 2750 | 1.81546e-05 |
| 2850 | 1.55051e-05 |
| 2950 | 5.51244e-06 |
| 3050 | 1.30865e-05 |
| 3150 | 1.3303e-05 |
| 3250 | 9.77694e-06 |
| 3350 | 1.50311e-05 |
| 3450 | 5.59385e-06 |
| 3550 | 5.42222e-06 |
| 3650 | 9.70567e-06 |
| 3750 | 2.19582e-06 |
| 3850 | 8.6116e-06 |
| 3950 | 6.58091e-06 |
| 4050 | 1.80993e-06 |
| 4150 | 8.59219e-06 |
| 4250 | 1.26502e-06 |
| 4350 | 0 |
| 4450 | 6.5388e-07 |
| 4550 | 3.72434e-06 |
| 4650 | 0 |
| 4750 | 3.15207e-06 |
| 4850 | 1.12194e-06 |
| 4950 | 0 |
| 5050 | 1.73905e-06 |
| 5150 | 1.58943e-06 |

a. 2000	b. 2010

**Figure 11 The curves of Getis-Ord’s *G* based on correlation density for the main cities of China**

(**Note**: Inside the scaling ranges of spatial correlation dimension, the curves of Getis-Ord’s *G* fluctuate significantly. From 2000 to 2010, the curve shapes have slight change.)

**[Reviewer #3]** Some other comments:

**[Reviewer #3]**1. Table 2”: it is better to introduce the unit of *r*, km in the table.

**[Reply]** This has been revised.

**[Reviewer #3]** 2. I think professional English editing is necessary to ensure the writing is of a high standard.

**[Reply]** Reviewer # 1 help me edit the manuscript thoroughly. Based on his/her edition, I revised the manuscript carefully.
