# Supplementary material for: An analytical process of spatial autocorrelation functions based on Moran’s index
Source: PLoS One. 2021 Apr 14;16(4):e0249589. doi: 10.1371/journal.pone.0249589 (PMC8046215; doi:10.1371/journal.pone.0249589)
Supplement: Attachment — Submitted filename: Response to Reviewers (R2)-2021-02-18.docx [file pone.0249589.s008.docx]

# Response to Reviewers (R2)

## Reviewer #1

**[Reviewer #1]** The paper has been improved, but suffers from a large number of minor language errors. I have left some substantial comments embedded in the PDF file, which I have uploaded here. There is one section where I would expect some citations for statements that I do not believe are correct.

**[Reply]** Thank you very much for checking out a series of clerical errors and mistakes in English expressions for me again.

**[Reviewer #1]** This is not really a fair statement, as there are many scaling methods and multiscale analyses. Perhaps less covered, but hardly ignored. (Page 2).

**[Reply]** I quite agree with you that there many scaling methods and multiscale analysis in the geographical literature and in the related fields. In the context of this paper, I discussed the problem of scaling idea absence in spatial autocorrelation analysis. Now, to avoid misunderstanding, the sentence has been revised as follows.

“First, in the theoretical aspect, the scaling property of geographical spatial autocorrelation has not been emphasized.”

**[Reviewer #1]** Also not true. Extensive work in kriging analysis has been done. PONE-D-20-34327R1
An Analytical Process of Spatial Autocorrelation Functions Based on Moran's Index
PLOS ONE

Dear Dr. Chen,

Thank you for submitting your manuscript to PLOS ONE. After careful consideration, we feel that it has merit but does not fully meet PLOS ONE’s publication criteria as it currently stands. Therefore, we invite you to submit a revised version of the manuscript that addresses the points raised during the review process. [Second, in the methodological aspect, the spatial displacement parameter has also been neglected. ] (Page 2).

Also not true. Extensive work in kriging analysis has been done.

**[Reply]** I am not familiar with Kriging analysis. Now, the sentence has been re-expressed as follows.

“Second, in the methodological aspect, the spatial displacement parameter (it is equivalent to the time lag parameter in time series analysis) has not been underlined in autocorrelation analyses. ” PONE-D-20-34327R1
An Analytical Process of Spatial Autocorrelation Functions Based on Moran's Index
PLOS ONE

Dear Dr. Chen,

Thank you for submitting your manuscript to PLOS ONE. After careful consideration, we feel that it has merit but does not fully meet PLOS ONE’s publication criteria as it currently stands. Therefore, we invite you to submit a revised version of the manuscript that addresses the points raised during the review process.

There are still some minor comments from two reviewers. I invite you to revise the manuscript accordingly.

Please submit your revised manuscript by Apr 04 2021 11:59PM. If you will need more time than this to complete your revisions, please reply to this message or contact the journal office at plosone@plos.org. When you're ready to submit your revision, log on to https://www.editorialmanager.com/pone/ and select the 'Submissions Needing Revision' folder to locate your manuscript file.

Please include the following items when submitting your revised manuscript:

- A rebuttal letter that responds to each point raised by the academic editor and reviewer(s). You should upload this letter as a separate file labeled 'Response to Reviewers'.
- A marked-up copy of your manuscript that highlights changes made to the original version. You should upload this as a separate file labeled 'Revised Manuscript with Track Changes'.
- An unmarked version of your revised paper without tracked changes. You should upload this as a separate file labeled 'Manuscript'.

If you would like to make changes to your financial disclosure, please include your updated statement in your cover letter. Guidelines for resubmitting your figure files are available below the reviewer comments at the end of this letter.

If applicable, we recommend that you deposit your laboratory protocols in protocols.io to enhance the reproducibility of your results. Protocols.io assigns your protocol its own identifier (DOI) so that it can be cited independently in the future. For instructions see: <http://journals.plos.org/plosone/s/submission-guidelines#loc-laboratory-protocols>

We look forward to receiving your revised manuscript.

Kind regards,

Bailang Yu
Academic Editor
PLOS ONE


Additional Editor Comments (if provided):

There are still some minor comments from two reviewers.


[Note: HTML markup is below. Please do not edit.]

Reviewers' comments:

Reviewer's Responses to Questions

**Comments to the Author**

1. If the authors have adequately addressed your comments raised in a previous round of review and you feel that this manuscript is now acceptable for publication, you may indicate that here to bypass the “Comments to the Author” section, enter your conflict of interest statement in the “Confidential to Editor” section, and submit your "Accept" recommendation.

Reviewer #1: (No Response)

Reviewer #3: All comments have been addressed

2. Is the manuscript technically sound, and do the data support the conclusions?

The manuscript must describe a technically sound piece of scientific research with data that supports the conclusions. Experiments must have been conducted rigorously, with appropriate controls, replication, and sample sizes. The conclusions must be drawn appropriately based on the data presented.

Reviewer #1: Yes

Reviewer #3: Yes

3. Has the statistical analysis been performed appropriately and rigorously?

Reviewer #1: Yes

Reviewer #3: Yes

4. Have the authors made all data underlying the findings in their manuscript fully available?

The [PLOS Data policy](http://www.plosone.org/static/policies.action#sharing) requires authors to make all data underlying the findings described in their manuscript fully available without restriction, with rare exception (please refer to the Data Availability Statement in the manuscript PDF file). The data should be provided as part of the manuscript or its supporting information, or deposited to a public repository. For example, in addition to summary statistics, the data points behind means, medians and variance measures should be available. If there are restrictions on publicly sharing data—e.g. participant privacy or use of data from a third party—those must be specified.

Reviewer #1: Yes

Reviewer #3: Yes

5. Is the manuscript presented in an intelligible fashion and written in standard English?

PLOS ONE does not copyedit accepted manuscripts, so the language in submitted articles must be clear, correct, and unambiguous. Any typographical or grammatical errors should be corrected at revision, so please note any specific errors here.

Reviewer #1: Yes

Reviewer #3: Yes

6. Review Comments to the Author

Please use the space provided to explain your answers to the questions above. You may also include additional comments for the author, including concerns about dual publication, research ethics, or publication ethics. (Please upload your review as an attachment if it exceeds 20,000 characters)

Reviewer #1: The paper has been improved, but suffers from a large number of minor language errors. I have left some substantial comments embedded in the PDF file, which I have uploaded here. There is one section where I would expect some citations for statements that I do not believe are correct.

Reviewer #3: Lines 556-558:"It is hard to clarify the whole questions about spatial scaling in the autocorrelation processes by means of several paragraph of words, and the related problems will be discussed in a companion paper."
Lines 693,694: "Due to limitation of space,...."
I don't think "space" can be a reason if the author does believe that the problem should be fully addressed in one paper. I would suggest tha author rephrase the sentence.

7. PLOS authors have the option to publish the peer review history of their article ([what does this mean?](https://journals.plos.org/plosone/s/editorial-and-peer-review-process#loc-peer-review-history)). If published, this will include your full peer review and any attached files.


If you choose “no”, your identity will remain anonymous but your review may still be made public.


**Do you want your identity to be public for this peer review?** For information about this choice, including consent withdrawal, please see our [Privacy Policy](https://www.plos.org/privacy-policy).

Reviewer #1: No

Reviewer #3: No

[NOTE: If reviewer comments were submitted as an attachment file, they will be attached to this email and accessible via the submission site. Please log into your account, locate the manuscript record, and check for the action link "View Attachments". If this link does not appear, there are no attachment files.]

While revising your submission, please upload your figure files to the Preflight Analysis and Conversion Engine (PACE) digital diagnostic tool, <https://pacev2.apexcovantage.com/>. PACE helps ensure that figures meet PLOS requirements. To use PACE, you must first register as a user. Registration is free. Then, login and navigate to the UPLOAD tab, where you will find detailed instructions on how to use the tool. If you encounter any issues or have any questions when using PACE, please email PLOS at [figures@plos.org](mailto:figures@plos.org). Please note that Supporting Information files do not need this step.

*In compliance with data protection regulations, you may request that we remove your personal registration details at any time.* [*(Remove my information/details)*](https://www.editorialmanager.com/pone/login.asp?a=r)*. Please contact the publication office if you have any questions.*PONE-D-20-34327R1
An Analytical Process of Spatial Autocorrelation Functions Based on Moran's Index
PLOS ONE

Dear Dr. Chen,

Thank you for submitting your manuscript to PLOS ONE. After careful consideration, we feel that it has merit but does not fully meet PLOS ONE’s publication criteria as it currently stands. Therefore, we invite you to submit a revised version of the manuscript that addresses the points raised during the review process.

There are still some minor comments from two reviewers. I invite you to revise the manuscript accordingly.

Please submit your revised manuscript by Apr 04 2021 11:59PM. If you will need more time than this to complete your revisions, please reply to this message or contact the journal office at plosone@plos.org. When you're ready to submit your revision, log on to https://www.editorialmanager.com/pone/ and select the 'Submissions Needing Revision' folder to locate your manuscript file.

Please include the following items when submitting your revised manuscript:

- A rebuttal letter that responds to each point raised by the academic editor and reviewer(s). You should upload this letter as a separate file labeled 'Response to Reviewers'.
- A marked-up copy of your manuscript that highlights changes made to the original version. You should upload this as a separate file labeled 'Revised Manuscript with Track Changes'.
- An unmarked version of your revised paper without tracked changes. You should upload this as a separate file labeled 'Manuscript'.

If you would like to make changes to your financial disclosure, please include your updated statement in your cover letter. Guidelines for resubmitting your figure files are available below the reviewer comments at the end of this letter.

If applicable, we recommend that you deposit your laboratory protocols in protocols.io to enhance the reproducibility of your results. Protocols.io assigns your protocol its own identifier (DOI) so that it can be cited independently in the future. For instructions see: <http://journals.plos.org/plosone/s/submission-guidelines#loc-laboratory-protocols>

We look forward to receiving your revised manuscript.

Kind regards,

Bailang Yu
Academic Editor
PLOS ONE


Additional Editor Comments (if provided):

There are still some minor comments from two reviewers.


[Note: HTML markup is below. Please do not edit.]

Reviewers' comments:

Reviewer's Responses to Questions

**Comments to the Author**

1. If the authors have adequately addressed your comments raised in a previous round of review and you feel that this manuscript is now acceptable for publication, you may indicate that here to bypass the “Comments to the Author” section, enter your conflict of interest statement in the “Confidential to Editor” section, and submit your "Accept" recommendation.

Reviewer #1: (No Response)

Reviewer #3: All comments have been addressed

2. Is the manuscript technically sound, and do the data support the conclusions?

The manuscript must describe a technically sound piece of scientific research with data that supports the conclusions. Experiments must have been conducted rigorously, with appropriate controls, replication, and sample sizes. The conclusions must be drawn appropriately based on the data presented.

Reviewer #1: Yes

Reviewer #3: Yes

3. Has the statistical analysis been performed appropriately and rigorously?

Reviewer #1: Yes

Reviewer #3: Yes

4. Have the authors made all data underlying the findings in their manuscript fully available?

The [PLOS Data policy](http://www.plosone.org/static/policies.action#sharing) requires authors to make all data underlying the findings described in their manuscript fully available without restriction, with rare exception (please refer to the Data Availability Statement in the manuscript PDF file). The data should be provided as part of the manuscript or its supporting information, or deposited to a public repository. For example, in addition to summary statistics, the data points behind means, medians and variance measures should be available. If there are restrictions on publicly sharing data—e.g. participant privacy or use of data from a third party—those must be specified.

Reviewer #1: Yes

Reviewer #3: Yes

5. Is the manuscript presented in an intelligible fashion and written in standard English?

PLOS ONE does not copyedit accepted manuscripts, so the language in submitted articles must be clear, correct, and unambiguous. Any typographical or grammatical errors should be corrected at revision, so please note any specific errors here.

Reviewer #1: Yes

Reviewer #3: Yes

6. Review Comments to the Author

Please use the space provided to explain your answers to the questions above. You may also include additional comments for the author, including concerns about dual publication, research ethics, or publication ethics. (Please upload your review as an attachment if it exceeds 20,000 characters)

Reviewer #1: The paper has been improved, but suffers from a large number of minor language errors. I have left some substantial comments embedded in the PDF file, which I have uploaded here. There is one section where I would expect some citations for statements that I do not believe are correct.

Reviewer #3: Lines 556-558:"It is hard to clarify the whole questions about spatial scaling in the autocorrelation processes by means of several paragraph of words, and the related problems will be discussed in a companion paper."
Lines 693,694: "Due to limitation of space,...."
I don't think "space" can be a reason if the author does believe that the problem should be fully addressed in one paper. I would suggest tha author rephrase the sentence.

7. PLOS authors have the option to publish the peer review history of their article ([what does this mean?](https://journals.plos.org/plosone/s/editorial-and-peer-review-process#loc-peer-review-history)). If published, this will include your full peer review and any attached files.


If you choose “no”, your identity will remain anonymous but your review may still be made public.


**Do you want your identity to be public for this peer review?** For information about this choice, including consent withdrawal, please see our [Privacy Policy](https://www.plos.org/privacy-policy).

Reviewer #1: No

Reviewer #3: No

[NOTE: If reviewer comments were submitted as an attachment file, they will be attached to this email and accessible via the submission site. Please log into your account, locate the manuscript record, and check for the action link "View Attachments". If this link does not appear, there are no attachment files.]

While revising your submission, please upload your figure files to the Preflight Analysis and Conversion Engine (PACE) digital diagnostic tool, <https://pacev2.apexcovantage.com/>. PACE helps ensure that figures meet PLOS requirements. To use PACE, you must first register as a user. Registration is free. Then, login and navigate to the UPLOAD tab, where you will find detailed instructions on how to use the tool. If you encounter any issues or have any questions when using PACE, please email PLOS at [figures@plos.org](mailto:figures@plos.org). Please note that Supporting Information files do not need this step.

**[Reviewer #1]** What are the blue lines? (Page 17)

**[Reply]** The blue lines represent the double values of the positive and negative standard error.

Now, a note has been added to one of the histograms as below:

“The blue lines in the histograms are termed ‘two-standard-error bands’, according to which we can know whether or not there is significant difference between ACF or PACF values and zero.”.PONE-D-20-34327R1
An Analytical Process of Spatial Autocorrelation Functions Based on Moran's Index
PLOS ONE

Dear Dr. Chen,

Thank you for submitting your manuscript to PLOS ONE. After careful consideration, we feel that it has merit but does not fully meet PLOS ONE’s publication criteria as it currently stands. Therefore, we invite you to submit a revised version of the manuscript that addresses the points raised during the review process.

There are still some minor comments from two reviewers. I invite you to revise the manuscript accordingly.

Please submit your revised manuscript by Apr 04 2021 11:59PM. If you will need more time than this to complete your revisions, please reply to this message or contact the journal office at plosone@plos.org. When you're ready to submit your revision, log on to https://www.editorialmanager.com/pone/ and select the 'Submissions Needing Revision' folder to locate your manuscript file.

Please include the following items when submitting your revised manuscript:

- A rebuttal letter that responds to each point raised by the academic editor and reviewer(s). You should upload this letter as a separate file labeled 'Response to Reviewers'.
- A marked-up copy of your manuscript that highlights changes made to the original version. You should upload this as a separate file labeled 'Revised Manuscript with Track Changes'.
- An unmarked version of your revised paper without tracked changes. You should upload this as a separate file labeled 'Manuscript'.

If you would like to make changes to your financial disclosure, please include your updated statement in your cover letter. Guidelines for resubmitting your figure files are available below the reviewer comments at the end of this letter.

If applicable, we recommend that you deposit your laboratory protocols in protocols.io to enhance the reproducibility of your results. Protocols.io assigns your protocol its own identifier (DOI) so that it can be cited independently in the future. For instructions see: <http://journals.plos.org/plosone/s/submission-guidelines#loc-laboratory-protocols>

We look forward to receiving your revised manuscript.

Kind regards,

Bailang Yu
Academic Editor
PLOS ONE


Additional Editor Comments (if provided):

There are still some minor comments from two reviewers.


[Note: HTML markup is below. Please do not edit.]

Reviewers' comments:

Reviewer's Responses to Questions

**Comments to the Author**

1. If the authors have adequately addressed your comments raised in a previous round of review and you feel that this manuscript is now acceptable for publication, you may indicate that here to bypass the “Comments to the Author” section, enter your conflict of interest statement in the “Confidential to Editor” section, and submit your "Accept" recommendation.

Reviewer #1: (No Response)

Reviewer #3: All comments have been addressed

2. Is the manuscript technically sound, and do the data support the conclusions?

The manuscript must describe a technically sound piece of scientific research with data that supports the conclusions. Experiments must have been conducted rigorously, with appropriate controls, replication, and sample sizes. The conclusions must be drawn appropriately based on the data presented.

Reviewer #1: Yes

Reviewer #3: Yes

3. Has the statistical analysis been performed appropriately and rigorously?

Reviewer #1: Yes

Reviewer #3: Yes

4. Have the authors made all data underlying the findings in their manuscript fully available?

The [PLOS Data policy](http://www.plosone.org/static/policies.action#sharing) requires authors to make all data underlying the findings described in their manuscript fully available without restriction, with rare exception (please refer to the Data Availability Statement in the manuscript PDF file). The data should be provided as part of the manuscript or its supporting information, or deposited to a public repository. For example, in addition to summary statistics, the data points behind means, medians and variance measures should be available. If there are restrictions on publicly sharing data—e.g. participant privacy or use of data from a third party—those must be specified.

Reviewer #1: Yes

Reviewer #3: Yes

5. Is the manuscript presented in an intelligible fashion and written in standard English?

PLOS ONE does not copyedit accepted manuscripts, so the language in submitted articles must be clear, correct, and unambiguous. Any typographical or grammatical errors should be corrected at revision, so please note any specific errors here.

Reviewer #1: Yes

Reviewer #3: Yes

6. Review Comments to the Author

Please use the space provided to explain your answers to the questions above. You may also include additional comments for the author, including concerns about dual publication, research ethics, or publication ethics. (Please upload your review as an attachment if it exceeds 20,000 characters)

Reviewer #1: The paper has been improved, but suffers from a large number of minor language errors. I have left some substantial comments embedded in the PDF file, which I have uploaded here. There is one section where I would expect some citations for statements that I do not believe are correct.

Reviewer #3: Lines 556-558:"It is hard to clarify the whole questions about spatial scaling in the autocorrelation processes by means of several paragraph of words, and the related problems will be discussed in a companion paper."
Lines 693,694: "Due to limitation of space,...."
I don't think "space" can be a reason if the author does believe that the problem should be fully addressed in one paper. I would suggest tha author rephrase the sentence.

7. PLOS authors have the option to publish the peer review history of their article ([what does this mean?](https://journals.plos.org/plosone/s/editorial-and-peer-review-process#loc-peer-review-history)). If published, this will include your full peer review and any attached files.


If you choose “no”, your identity will remain anonymous but your review may still be made public.


**Do you want your identity to be public for this peer review?** For information about this choice, including consent withdrawal, please see our [Privacy Policy](https://www.plos.org/privacy-policy).

Reviewer #1: No

Reviewer #3: No

[NOTE: If reviewer comments were submitted as an attachment file, they will be attached to this email and accessible via the submission site. Please log into your account, locate the manuscript record, and check for the action link "View Attachments". If this link does not appear, there are no attachment files.]

While revising your submission, please upload your figure files to the Preflight Analysis and Conversion Engine (PACE) digital diagnostic tool, <https://pacev2.apexcovantage.com/>. PACE helps ensure that figures meet PLOS requirements. To use PACE, you must first register as a user. Registration is free. Then, login and navigate to the UPLOAD tab, where you will find detailed instructions on how to use the tool. If you encounter any issues or have any questions when using PACE, please email PLOS at [figures@plos.org](mailto:figures@plos.org). Please note that Supporting Information files do not need this step.

*In compliance with data protection regulations, you may request that we remove your personal registration details at any time.* [*(Remove my information/details)*](https://www.editorialmanager.com/pone/login.asp?a=r)*. Please contact the publication office if you have any questions.*PONE-D-20-34327R1
An Analytical Process of Spatial Autocorrelation Functions Based on Moran's Index
PLOS ONE

Dear Dr. Chen,

Thank you for submitting your manuscript to PLOS ONE. After careful consideration, we feel that it has merit but does not fully meet PLOS ONE’s publication criteria as it currently stands. Therefore, we invite you to submit a revised version of the manuscript that addresses the points raised during the review process.

There are still some minor comments from two reviewers. I invite you to revise the manuscript accordingly.

Please submit your revised manuscript by Apr 04 2021 11:59PM. If you will need more time than this to complete your revisions, please reply to this message or contact the journal office at plosone@plos.org. When you're ready to submit your revision, log on to https://www.editorialmanager.com/pone/ and select the 'Submissions Needing Revision' folder to locate your manuscript file.

Please include the following items when submitting your revised manuscript:

- A rebuttal letter that responds to each point raised by the academic editor and reviewer(s). You should upload this letter as a separate file labeled 'Response to Reviewers'.
- A marked-up copy of your manuscript that highlights changes made to the original version. You should upload this as a separate file labeled 'Revised Manuscript with Track Changes'.
- An unmarked version of your revised paper without tracked changes. You should upload this as a separate file labeled 'Manuscript'.

If you would like to make changes to your financial disclosure, please include your updated statement in your cover letter. Guidelines for resubmitting your figure files are available below the reviewer comments at the end of this letter.

If applicable, we recommend that you deposit your laboratory protocols in protocols.io to enhance the reproducibility of your results. Protocols.io assigns your protocol its own identifier (DOI) so that it can be cited independently in the future. For instructions see: <http://journals.plos.org/plosone/s/submission-guidelines#loc-laboratory-protocols>

We look forward to receiving your revised manuscript.

Kind regards,

Bailang Yu
Academic Editor
PLOS ONE


Additional Editor Comments (if provided):

There are still some minor comments from two reviewers.


[Note: HTML markup is below. Please do not edit.]

Reviewers' comments:

Reviewer's Responses to Questions

**Comments to the Author**

1. If the authors have adequately addressed your comments raised in a previous round of review and you feel that this manuscript is now acceptable for publication, you may indicate that here to bypass the “Comments to the Author” section, enter your conflict of interest statement in the “Confidential to Editor” section, and submit your "Accept" recommendation.

Reviewer #1: (No Response)

Reviewer #3: All comments have been addressed

2. Is the manuscript technically sound, and do the data support the conclusions?

The manuscript must describe a technically sound piece of scientific research with data that supports the conclusions. Experiments must have been conducted rigorously, with appropriate controls, replication, and sample sizes. The conclusions must be drawn appropriately based on the data presented.

Reviewer #1: Yes

Reviewer #3: Yes

3. Has the statistical analysis been performed appropriately and rigorously?

Reviewer #1: Yes

Reviewer #3: Yes

4. Have the authors made all data underlying the findings in their manuscript fully available?

The [PLOS Data policy](http://www.plosone.org/static/policies.action#sharing) requires authors to make all data underlying the findings described in their manuscript fully available without restriction, with rare exception (please refer to the Data Availability Statement in the manuscript PDF file). The data should be provided as part of the manuscript or its supporting information, or deposited to a public repository. For example, in addition to summary statistics, the data points behind means, medians and variance measures should be available. If there are restrictions on publicly sharing data—e.g. participant privacy or use of data from a third party—those must be specified.

Reviewer #1: Yes

Reviewer #3: Yes

5. Is the manuscript presented in an intelligible fashion and written in standard English?

PLOS ONE does not copyedit accepted manuscripts, so the language in submitted articles must be clear, correct, and unambiguous. Any typographical or grammatical errors should be corrected at revision, so please note any specific errors here.

Reviewer #1: Yes

Reviewer #3: Yes

6. Review Comments to the Author

Please use the space provided to explain your answers to the questions above. You may also include additional comments for the author, including concerns about dual publication, research ethics, or publication ethics. (Please upload your review as an attachment if it exceeds 20,000 characters)

Reviewer #1: The paper has been improved, but suffers from a large number of minor language errors. I have left some substantial comments embedded in the PDF file, which I have uploaded here. There is one section where I would expect some citations for statements that I do not believe are correct.

Reviewer #3: Lines 556-558:"It is hard to clarify the whole questions about spatial scaling in the autocorrelation processes by means of several paragraph of words, and the related problems will be discussed in a companion paper."
Lines 693,694: "Due to limitation of space,...."
I don't think "space" can be a reason if the author does believe that the problem should be fully addressed in one paper. I would suggest tha author rephrase the sentence.

7. PLOS authors have the option to publish the peer review history of their article ([what does this mean?](https://journals.plos.org/plosone/s/editorial-and-peer-review-process#loc-peer-review-history)). If published, this will include your full peer review and any attached files.


If you choose “no”, your identity will remain anonymous but your review may still be made public.


**Do you want your identity to be public for this peer review?** For information about this choice, including consent withdrawal, please see our [Privacy Policy](https://www.plos.org/privacy-policy).

Reviewer #1: No

Reviewer #3: No

[NOTE: If reviewer comments were submitted as an attachment file, they will be attached to this email and accessible via the submission site. Please log into your account, locate the manuscript record, and check for the action link "View Attachments". If this link does not appear, there are no attachment files.]

While revising your submission, please upload your figure files to the Preflight Analysis and Conversion Engine (PACE) digital diagnostic tool, <https://pacev2.apexcovantage.com/>. PACE helps ensure that figures meet PLOS requirements. To use PACE, you must first register as a user. Registration is free. Then, login and navigate to the UPLOAD tab, where you will find detailed instructions on how to use the tool. If you encounter any issues or have any questions when using PACE, please email PLOS at [figures@plos.org](mailto:figures@plos.org). Please note that Supporting Information files do not need this step.

“The significance of the autocorrelation function can be judged by the double values of positive and negative standard errors. The standard error value can be estimated with the reciprocal of the square root of the number of sampling points (Diebold, 2007). For spatial autocorrelation, if *n* geographical elements are taken into account, the maximum number of sampling points can be treated as *n*^2^, and thus, based on the significance level *α*=0.05, the standard errors can is about 1/*n*.”

Diebold FX (2007). *Elements of Forecasting* (4^th^ ed.). Mason, Ohio: Thomson/South-Western

**[Reviewer #1]** These two plots could be superimposed at the same vertical scale. (Page 21).

**[Reply]** The two subplots have been replaced by the following plot.

SACF/PSACF

| Distance (km) | AP2000 | AP2010 |
| --- | --- | --- |
| 150 | 1 | 1 |
| 250 | 0.987163 | 1.0402 |
| 350 | 1.00356 | 0.99185 |
| 450 | 1.00594 | 1.00923 |
| 550 | 1.01138 | 1.01085 |
| 650 | 0.999364 | 1.09467 |
| 750 | 0.996932 | 1.00218 |
| 850 | 0.99723 | 1.00223 |
| 950 | 0.98645 | 0.964036 |
| 1050 | 0.99252 | 0.994935 |
| 1150 | 0.983387 | 1.29259 |
| 1250 | 1.01025 | 1.01579 |
| 1350 | 0.982553 | 0.956449 |
| 1450 | 0.970283 | 0.992398 |
| 1550 | 0.999723 | 1.00442 |
| 1650 | 0.970053 | 0.941519 |
| 1750 | 1.02312 | 1.00133 |
| 1850 | 1.0002 | 1.0193 |
| 1950 | 1.00485 | 1.01762 |
| 2050 | 0.983531 | 0.987448 |
| 2150 | 0.990247 | 0.98546 |
| 2250 | 1.03457 | 1.02963 |
| 2350 | 0.987238 | 0.962038 |
| 2450 | 0.963988 | 0.943495 |
| 2550 | 0.959253 | 0.956384 |
| 2650 | 0.963559 | 0.961333 |
| 2750 | 0.876968 | 0.939064 |
| 2850 | 1.52453 | 1.31972 |
| 2950 | 20.8018 | 1.40649 |
| 3050 | 0.91738 | 0.937404 |
| 3150 | 0.883678 | 0.946865 |
| 3250 | 1.14227 | 1.13983 |
| 3350 | 0.837497 | 0.844601 |
| 3450 | 0.025027 | 0.00335785 |
| 3550 | 0.559012 | 0.660325 |
| 3650 | 7.53555 | 0.369177 |
| 3750 | 1.09268 | 0.872146 |
| 3850 | 0.82717 | 0.799678 |
| 3950 | 3.7986 | 2.81863 |
| 4050 | 1.67564 | 1.65083 |
| 4150 | 1.03137 | 1.05513 |
| 4250 | 0.255311 | 0.480735 |
| 4350 | 0 | 0 |
| 4450 | 0.664014 | 0.746813 |
| 4550 | 1.12691 | 10.1537 |
| 4650 | 0 | 0 |
| 4750 | 1.15344 | 1.21877 |
| 4850 | -3.68104 | -11.5464 |
| 4950 | 0 | 0 |
| 5050 | 0.976624 | 0.958591 |
| 5150 | 0.079618 | -0.413625 |

**Figure 6 The ratios of SACF to PSACF based on correlation density for the main cities of China**

**[Reviewer #1]** One would expect reverse spatial autocorrelation, as big cities are more likely further from other large cities, not closer. (Page 22).

**[Reply]** Now, the sentence “Therefore, on the national dimension, population migration between large cities is not easy” has been revised as follows: “Therefore, on the national dimension, population migration between large cities is not very free.”

Spatial autocorrelation proved to be consistent with spatial potential which is based on gravity model (Chen, 2020). The spatial mobility of population is significantly affected by two factors: one is spatial distance, and the other is city sizes. The influence of distance on population mobility is greater than that of city sizes. Based on the data of the fifth population census, China's population mobility models are as follows

$T_{ij}=0.001894\frac{P_{i}^{0.6366}P_{j}^{0.8067}}{r_{ij}^{1.1628}}$,$T_{ij}=0.001894\frac{P_{i}^{0.8067}P_{j}^{0.6366}}{r_{ij}^{1.1628}}$.

The scaling exponents of city sizes are 0.6366 and 8067, which are less than 1; the absolute value of the distance scaling exponent is 1.1628, which is greater than 1. Based on the data of the sixth population census, China's population mobility models are as follows

$T_{ij}=0.0161\frac{P_{i}^{0.6713}P_{j}^{0.6381}}{r_{ij}^{1.0506}}$,$T_{ji}=0.0161\frac{P_{i}^{0.6381}P_{j}^{0.6713}}{r_{ij}^{1.0506}}$.

The scaling exponents of city sizes are 0.6713 and 0.6381, which are less than 1; the absolute value of the distance scaling exponent is 1.0506, which is greater than 1. According to the scaling exponent values, we can inference that the influence of distance on population mobility is greater than that of city sizes.

- Chen YG (2020). New framework of Getis-Ord’s indexes associating spatial autocorrelation with interaction. *PLoS ONE*, 15(7): e0236765

**[Reviewer #1]** I don't see how this conclusion about population size and travel distance relates to migration. (Page 22).

**[Reply]** There are two grounds for the judgment:

First, the correlogram based on zero diagonal weight matrix (self-correlation of each city is not taken into account) differ significantly from that based on nonzero diagonal weight matrix (self-correlation of each city is taken into account).

Second, if the diagonal elements are taken into account, there is no significant difference between the spatial ACF and to the spatial PACF.

**[Reviewer #1]** I don't understand what you mean by this sentence.[“However, the substantial research on spatial autocorrelation based on scaling has not been reported.”]. (Page 30).

**[Reply]** To avoid misunderstanding, the sentence has been revised as blow:

“However, the substantial research on scaling in spatial autocorrelation process has not been reported.”

**[Reviewer #1]** However, you only show then with a single dimension. (Page 30).

**[Reply]** The 2-dimensional geographical phenomena can be described from 1-dimensional angle of view or 2-dimensional angle of view. For example, the well-known Clark’s model of urban population density is to describe 2-dimensional space phenomenon from the perspective of 1-dimensional space based on statistical average. Based on Clark’s model, the 1-dimensional spatial autocorrelation function can be constructed easily.

One dimension space description involves an independent direction, while two dimension space description requires two orthogonal directions. The spatial autocorrelation functions defined in this paper are based on two orthogonal directions.

The connection and difference between the 1-dimensional spatial autocorrelation and the 2-dimensional spatial autocorrelation have been illustrated in a previous paper as follows (Chen, 2019).

**Figure 1 The sketch maps of three types of spatial correlation processes**

**Note:** As schematic diagrams, only five urban elements are taken into account. The concentric circles are employed to compute average density of urban land use or traffic networks. The space between two immediate large circles forms a ring, the small circles fall between two circles represent cells along a radial line, and the arcs indicate spatial correlation between two cells. Subgraphs: (a) For the 2-dimension spatial correlation, we need five sets of concentric circles to determine spatial scaling; (b) For the 1-dimension spatial correlation, we need one set of concentric circles to compute average density; (c) The point-point correlation (1-dimension correlation) suggests density-density correlation; (d) The one-point correlation (1-dimension correlation) suggests central correlation.

- Chen YG (2019). Fractal dimension analysis of urban morphology based on spatial correlation functions. In: D'Acci L(ed.). *Mathematics of Urban Morphology*. Birkhäuser: Springer Nature Switzerland AG, pp21-53

Table 3 are revised as follows.

**Table 3 A comparison of the analytical processes of autocorrelation functions and related methods**

| Domain | Dimension | Object | ACF | PACF | Spectral analysis |
| --- | --- | --- | --- | --- | --- |
| Time domain | 1-dimension time (one independent direction) | Time series | Temporal ACF analysis and auto-regressive process | Temporal PACF analysis and auto-regressive process | Power spectrum |
| Spatial domain | 1-dimension space (one independent direction) | Isotropic ordered spatial series | Spatial ACF analysis and auto-regressive process | Spatial PACF analysis and auto-regressive process | Wave spectrum |
|  | 2-dimension space (two independent directions) | Anisotropic random spatial series | To be developed | To be developed | To be developed |

## Reviewer #2

**[Reviewer #3]** Lines 556-558: "It is hard to clarify the whole questions about spatial scaling in the autocorrelation processes by means of several paragraph of words, and the related problems will be discussed in a companion paper."

Lines 693,694: "Due to limitation of space,...."

I don't think "space" can be a reason if the author does believe that the problem should be fully addressed in one paper. I would suggest that author rephrase the sentence.

**[Reply]** These type of expressions have been revised or deleted. Thanks.PONE-D-20-34327R1
An Analytical Process of Spatial Autocorrelation Functions Based on Moran's Index
PLOS ONE

Dear Dr. Chen,

Thank you for submitting your manuscript to PLOS ONE. After careful consideration, we feel that it has merit but does not fully meet PLOS ONE’s publication criteria as it currently stands. Therefore, we invite you to submit a revised version of the manuscript that addresses the points raised during the review process.

There are still some minor comments from two reviewers. I invite you to revise the manuscript accordingly.

Please submit your revised manuscript by Apr 04 2021 11:59PM. If you will need more time than this to complete your revisions, please reply to this message or contact the journal office at plosone@plos.org. When you're ready to submit your revision, log on to https://www.editorialmanager.com/pone/ and select the 'Submissions Needing Revision' folder to locate your manuscript file.

Please include the following items when submitting your revised manuscript:

- A rebuttal letter that responds to each point raised by the academic editor and reviewer(s). You should upload this letter as a separate file labeled 'Response to Reviewers'.
- A marked-up copy of your manuscript that highlights changes made to the original version. You should upload this as a separate file labeled 'Revised Manuscript with Track Changes'.
- An unmarked version of your revised paper without tracked changes. You should upload this as a separate file labeled 'Manuscript'.

If you would like to make changes to your financial disclosure, please include your updated statement in your cover letter. Guidelines for resubmitting your figure files are available below the reviewer comments at the end of this letter.

If applicable, we recommend that you deposit your laboratory protocols in protocols.io to enhance the reproducibility of your results. Protocols.io assigns your protocol its own identifier (DOI) so that it can be cited independently in the future. For instructions see: <http://journals.plos.org/plosone/s/submission-guidelines#loc-laboratory-protocols>

We look forward to receiving your revised manuscript.

Kind regards,

Bailang Yu
Academic Editor
PLOS ONE


Additional Editor Comments (if provided):

There are still some minor comments from two reviewers.


[Note: HTML markup is below. Please do not edit.]

Reviewers' comments:

Reviewer's Responses to Questions

**Comments to the Author**

1. If the authors have adequately addressed your comments raised in a previous round of review and you feel that this manuscript is now acceptable for publication, you may indicate that here to bypass the “Comments to the Author” section, enter your conflict of interest statement in the “Confidential to Editor” section, and submit your "Accept" recommendation.

Reviewer #1: (No Response)

Reviewer #3: All comments have been addressed

2. Is the manuscript technically sound, and do the data support the conclusions?

The manuscript must describe a technically sound piece of scientific research with data that supports the conclusions. Experiments must have been conducted rigorously, with appropriate controls, replication, and sample sizes. The conclusions must be drawn appropriately based on the data presented.

Reviewer #1: Yes

Reviewer #3: Yes

3. Has the statistical analysis been performed appropriately and rigorously?

Reviewer #1: Yes

Reviewer #3: Yes

4. Have the authors made all data underlying the findings in their manuscript fully available?

The [PLOS Data policy](http://www.plosone.org/static/policies.action#sharing) requires authors to make all data underlying the findings described in their manuscript fully available without restriction, with rare exception (please refer to the Data Availability Statement in the manuscript PDF file). The data should be provided as part of the manuscript or its supporting information, or deposited to a public repository. For example, in addition to summary statistics, the data points behind means, medians and variance measures should be available. If there are restrictions on publicly sharing data—e.g. participant privacy or use of data from a third party—those must be specified.

Reviewer #1: Yes

Reviewer #3: Yes

5. Is the manuscript presented in an intelligible fashion and written in standard English?

PLOS ONE does not copyedit accepted manuscripts, so the language in submitted articles must be clear, correct, and unambiguous. Any typographical or grammatical errors should be corrected at revision, so please note any specific errors here.

Reviewer #1: Yes

Reviewer #3: Yes

6. Review Comments to the Author

Please use the space provided to explain your answers to the questions above. You may also include additional comments for the author, including concerns about dual publication, research ethics, or publication ethics. (Please upload your review as an attachment if it exceeds 20,000 characters)

Reviewer #1: The paper has been improved, but suffers from a large number of minor language errors. I have left some substantial comments embedded in the PDF file, which I have uploaded here. There is one section where I would expect some citations for statements that I do not believe are correct.

Reviewer #3: Lines 556-558:"It is hard to clarify the whole questions about spatial scaling in the autocorrelation processes by means of several paragraph of words, and the related problems will be discussed in a companion paper."
Lines 693,694: "Due to limitation of space,...."
I don't think "space" can be a reason if the author does believe that the problem should be fully addressed in one paper. I would suggest tha author rephrase the sentence.

7. PLOS authors have the option to publish the peer review history of their article ([what does this mean?](https://journals.plos.org/plosone/s/editorial-and-peer-review-process#loc-peer-review-history)). If published, this will include your full peer review and any attached files.


If you choose “no”, your identity will remain anonymous but your review may still be made public.


**Do you want your identity to be public for this peer review?** For information about this choice, including consent withdrawal, please see our [Privacy Policy](https://www.plos.org/privacy-policy).

Reviewer #1: No

Reviewer #3: No

[NOTE: If reviewer comments were submitted as an attachment file, they will be attached to this email and accessible via the submission site. Please log into your account, locate the manuscript record, and check for the action link "View Attachments". If this link does not appear, there are no attachment files.]

While revising your submission, please upload your figure files to the Preflight Analysis and Conversion Engine (PACE) digital diagnostic tool, <https://pacev2.apexcovantage.com/>. PACE helps ensure that figures meet PLOS requirements. To use PACE, you must first register as a user. Registration is free. Then, login and navigate to the UPLOAD tab, where you will find detailed instructions on how to use the tool. If you encounter any issues or have any questions when using PACE, please email PLOS at [figures@plos.org](mailto:figures@plos.org). Please note that Supporting Information files do not need this step.

*In compliance with data protection regulations, you may request that we remove your personal registration details at any time.* [*(Remove my information/details)*](https://www.editorialmanager.com/pone/login.asp?a=r)*. Please contact the publication office if you have any questions.*PONE-D-20-34327R1
An Analytical Process of Spatial Autocorrelation Functions Based on Moran's Index
PLOS ONE

Dear Dr. Chen,

Thank you for submitting your manuscript to PLOS ONE. After careful consideration, we feel that it has merit but does not fully meet PLOS ONE’s publication criteria as it currently stands. Therefore, we invite you to submit a revised version of the manuscript that addresses the points raised during the review process.

There are still some minor comments from two reviewers. I invite you to revise the manuscript accordingly.

Please submit your revised manuscript by Apr 04 2021 11:59PM. If you will need more time than this to complete your revisions, please reply to this message or contact the journal office at plosone@plos.org. When you're ready to submit your revision, log on to https://www.editorialmanager.com/pone/ and select the 'Submissions Needing Revision' folder to locate your manuscript file.

Please include the following items when submitting your revised manuscript:

- A rebuttal letter that responds to each point raised by the academic editor and reviewer(s). You should upload this letter as a separate file labeled 'Response to Reviewers'.
- A marked-up copy of your manuscript that highlights changes made to the original version. You should upload this as a separate file labeled 'Revised Manuscript with Track Changes'.
- An unmarked version of your revised paper without tracked changes. You should upload this as a separate file labeled 'Manuscript'.

If you would like to make changes to your financial disclosure, please include your updated statement in your cover letter. Guidelines for resubmitting your figure files are available below the reviewer comments at the end of this letter.

If applicable, we recommend that you deposit your laboratory protocols in protocols.io to enhance the reproducibility of your results. Protocols.io assigns your protocol its own identifier (DOI) so that it can be cited independently in the future. For instructions see: <http://journals.plos.org/plosone/s/submission-guidelines#loc-laboratory-protocols>

We look forward to receiving your revised manuscript.

Kind regards,

Bailang Yu
Academic Editor
PLOS ONE


Additional Editor Comments (if provided):

There are still some minor comments from two reviewers.


[Note: HTML markup is below. Please do not edit.]

Reviewers' comments:

Reviewer's Responses to Questions

**Comments to the Author**

1. If the authors have adequately addressed your comments raised in a previous round of review and you feel that this manuscript is now acceptable for publication, you may indicate that here to bypass the “Comments to the Author” section, enter your conflict of interest statement in the “Confidential to Editor” section, and submit your "Accept" recommendation.

Reviewer #1: (No Response)

Reviewer #3: All comments have been addressed

2. Is the manuscript technically sound, and do the data support the conclusions?

The manuscript must describe a technically sound piece of scientific research with data that supports the conclusions. Experiments must have been conducted rigorously, with appropriate controls, replication, and sample sizes. The conclusions must be drawn appropriately based on the data presented.

Reviewer #1: Yes

Reviewer #3: Yes

3. Has the statistical analysis been performed appropriately and rigorously?

Reviewer #1: Yes

Reviewer #3: Yes

4. Have the authors made all data underlying the findings in their manuscript fully available?

The [PLOS Data policy](http://www.plosone.org/static/policies.action#sharing) requires authors to make all data underlying the findings described in their manuscript fully available without restriction, with rare exception (please refer to the Data Availability Statement in the manuscript PDF file). The data should be provided as part of the manuscript or its supporting information, or deposited to a public repository. For example, in addition to summary statistics, the data points behind means, medians and variance measures should be available. If there are restrictions on publicly sharing data—e.g. participant privacy or use of data from a third party—those must be specified.

Reviewer #1: Yes

Reviewer #3: Yes

5. Is the manuscript presented in an intelligible fashion and written in standard English?

PLOS ONE does not copyedit accepted manuscripts, so the language in submitted articles must be clear, correct, and unambiguous. Any typographical or grammatical errors should be corrected at revision, so please note any specific errors here.

Reviewer #1: Yes

Reviewer #3: Yes

6. Review Comments to the Author

Please use the space provided to explain your answers to the questions above. You may also include additional comments for the author, including concerns about dual publication, research ethics, or publication ethics. (Please upload your review as an attachment if it exceeds 20,000 characters)

Reviewer #1: The paper has been improved, but suffers from a large number of minor language errors. I have left some substantial comments embedded in the PDF file, which I have uploaded here. There is one section where I would expect some citations for statements that I do not believe are correct.

Reviewer #3: Lines 556-558:"It is hard to clarify the whole questions about spatial scaling in the autocorrelation processes by means of several paragraph of words, and the related problems will be discussed in a companion paper."
Lines 693,694: "Due to limitation of space,...."
I don't think "space" can be a reason if the author does believe that the problem should be fully addressed in one paper. I would suggest tha author rephrase the sentence.

7. PLOS authors have the option to publish the peer review history of their article ([what does this mean?](https://journals.plos.org/plosone/s/editorial-and-peer-review-process#loc-peer-review-history)). If published, this will include your full peer review and any attached files.


If you choose “no”, your identity will remain anonymous but your review may still be made public.


**Do you want your identity to be public for this peer review?** For information about this choice, including consent withdrawal, please see our [Privacy Policy](https://www.plos.org/privacy-policy).

Reviewer #1: No

Reviewer #3: No

[NOTE: If reviewer comments were submitted as an attachment file, they will be attached to this email and accessible via the submission site. Please log into your account, locate the manuscript record, and check for the action link "View Attachments". If this link does not appear, there are no attachment files.]

While revising your submission, please upload your figure files to the Preflight Analysis and Conversion Engine (PACE) digital diagnostic tool, <https://pacev2.apexcovantage.com/>. PACE helps ensure that figures meet PLOS requirements. To use PACE, you must first register as a user. Registration is free. Then, login and navigate to the UPLOAD tab, where you will find detailed instructions on how to use the tool. If you encounter any issues or have any questions when using PACE, please email PLOS at [figures@plos.org](mailto:figures@plos.org). Please note that Supporting Information files do not need this step.
